# The hypoxia-induced chromatin reader ZMYND8 drives HIF-dependent metabolic rewiring in breast cancer

**DOI:** 10.1016/j.jbc.2025.110680

**Published:** 2025-09-03

**Authors:** Sandhik Nandi, Atanu Mondal, Ishita Sarkar, Md Wasim Akram Ddoza Hazari, Indrakshi Banerjee, Shantanu Ghosh, Himansu Roy, Abhra Banerjee, Anjali Bandyopadhyay, Shritama Aich, Sanghamitra Sengupta, Shilpak Chatterjee, Chandrima Das

**Affiliations:** 1 Biophysics and Structural Genomics Division, Saha Institute of Nuclear Physics, Kolkata, India; 2 Homi Bhabha National Institute, Mumbai, India; 3 Division of Cancer Biology and Inflammatory Disorder, IICB-Translational Research Unit of Excellence, CSIR–Indian Institute of Chemical Biology, Kolkata, India; 4 Academy of Scientific and Innovative Research (AcSIR), Ghaziabad, India; 5 Department of Biochemistry, University of Calcutta, Kolkata, West Bengal, India; 6 Department of Surgery, KPC Medical College and Hospital, Kolkata, West Bengal, India; 7 Multi-Disciplinary Research Unit, R.G.Kar Medical College and Hospital, Kolkata, West Bengal, India; 8 Department of Pathology, R.G.Kar Medical College and Hospital, Kolkata, West Bengal, India

**Keywords:** chromatin, gene expression, transcription, breast cancer, hypoxia, glucose metabolism, glycolysis, lactate, immune signaling

## Abstract

Breast cancer, a leading cause of mortality, exhibits significant heterogeneity across molecular subtypes, with tumor hypoxia contributing to poor therapeutic outcomes. The present study investigates the role of ZMYND8, a hypoxia-responsive epigenetic factor, in regulating carbohydrate metabolism in concert with HIF1α in breast cancer. In adherent cells as well as in 3D MCTS, ZMYND8 expression is elevated under hypoxic conditions. Furthermore, immunohistochemistry analysis also shows that ZMYND8 and HIF1α expression are positively correlated in breast cancer. Remarkably, ZMYND8 is found to regulate glycolysis in hypoxic breast cancer cells as well as in 4T1-induced breast tumors in mice, elevating the expression of hexokinase II (*HKII*) and lactate dehydrogenase A. Notably, ZMYND8 directly regulates the transcription of lactate dehydrogenase A by promoting the recruitment of S5-phosphorylated RNA polymerase II to its promoter region. Metabolic-flux analysis, along with acetyl CoA and lactate pool measurements confirm that ZMYND8 bifurcate the metabolic axis toward anaerobic glycolysis, leading to the increase of extracellular acidification in hypoxic conditions. Interestingly, ZMYND8-induced changes in metabolic intermediate lactate in breast cancer cells, as well as in mouse serum, significantly impact the immune cell invasion and CD8+ T cell activity in the tumor microenvironment. These results highlight ZMYND8 as a key player in hypoxia-induced metabolic reprogramming of breast cancer cells and provide new insights into the epigenetic regulation of cancer metabolism. Our study unveils a novel mechanism linking epigenetics, metabolism, and immune evasion in breast cancer, opening new avenues for targeted therapeutic interventions aimed at disrupting this axis.

The epigenome stands as a crucial regulator of cellular physiology, orchestrating a complex array of mechanisms that govern gene expression and cellular function. Epigenetic reprogramming plays a fundamental role in the maintenance of normal cellular functions, ensuring cell-type-specific gene expression patterns, which are crucial for the development of complex organisms (1). The dynamic and reversible nature of epigenetic modifications, encompassing changes in DNA methylation, histone posttranslational modifications, and noncoding RNA-mediated regulation of gene expression, has increasingly been associated with human diseases, most notably cancer (2, 3, 4). Indeed, epigenetic reprogramming is now recognized as one of the hallmarks of cancer progression, underscoring its significance in both normal physiology and pathological states.

Zinc finger MYND-type containing 8 (ZMYND8) has emerged as a key epigenetic player, demonstrating a diverse range of functions in transcriptional regulation (5). ZMYND8 is intricately involved in several critical cellular processes, including its role as a component of the elongating RNA polymerase complex through its association with BRD4 (6, 7). This protein is also responsible for super-enhancer mediated transcription regulation (8) highlighting its importance in controlling gene expression on a broader scale. Intriguingly, ZMYND8 functions as a dual histone reader of H3.1K36me2/H4K16ac, regulating ATRA-mediated transcription regulation (7). It also regulates epithelial–mesenchymal transition programs (9) and is endowed with an ability to regulate the terminal differentiation programs in breast cancer through its chromatin-binding function (10). In breast cancer, ZMYND8 is reported to be a coactivator of hypoxia-inducible factor (HIF) target genes and itself gets acetylated and regulates metastasis (11, 12). This connection to HIFs is particularly significant given the critical role of hypoxia in tumor progression and metastasis (13). Moreover, ZMYND8 has been shown to positively regulate the hexokinase II (HK II) gene by recruiting BRD4 to its promoter, thereby supporting elevated glycolysis and cellular proliferation (14, 15). In addition, ZMYND8 is elevated in breast cancer stem cells (BCSCs), where it enhances the biosynthesis of 27-hydroxycholesterol (27-HC) and suppresses its breakdown, leading to the oncogenic transformation of breast cancer stem cells and the tumor initiation (16).

The recognition of metabolic adaptation as a hallmark of cancer (17) has opened new avenues for understanding tumor biology and developing targeted therapies. Cancer cells exhibit a remarkable ability to reprogram their metabolism to support rapid proliferation, survival under stress conditions, and metastasis (18). This metabolic rewiring, often involving augmented glycolysis, in normoxic conditions, is commonly known as the Warburg effect (17, 19, 20). However, the metabolic landscape of tumors is far more complex than initially believed, with different regions of solid tumors exhibiting distinct metabolic profiles based on their microenvironment.

The tumor microenvironment plays a crucial role in shaping cancer cell metabolism (21). Despite the process of angiogenesis, which aims to supply tumors with blood vessels for nutrient and oxygen delivery, the rapid and uncontrolled growth of solid tumors often outpaces vascularization (13, 22, 23, 24). This leads to the formation of inner hypoxic regions characterized by limited oxygen tension (13, 22). These hypoxic regions of solid tumors display discrete phenotypic, metabolic, and molecular signatures that are distinct from normoxic tumor mass (25, 26).

The hypoxic tumor microenvironment comprises a diverse array of immune cells, including T cells, macrophages, dendritic cells, and natural killer cells (13). T cells are a major contributor to antitumor immunity (27). The effectiveness of CD8+ T cells primarily determines the outcome of antitumor response (27). Hypoxic tension in the tumor microenvironment generates an acidic tumor milieu (13). Lactate in the hypoxic milieu has been reported to neutralize the effectiveness of T cells, leading to dampening of antitumor immunity (28). Understanding the metabolic adaptations that enable cancer cells to survive and proliferate, thereby evading the immune response under nutrient- and oxygen-limited conditions, is a critical area of cancer research.

In response to hypoxia, cancer cells activate complex adaptive mechanisms, primarily mediated by hypoxia-inducible factor 1-α (HIF1α) (29). These adaptations include increased glycolysis along with attenuated oxidative phosphorylation. However, this augmented glycolytic flux in core hypoxic tumor cells presents a paradox, given the severely reduced availability of glucose in these regions. How these core hypoxic cells sustain their energy demands has remained elusive. A few studies have indicated that hypoxic cancer cells show an abbreviated form of gluconeogenesis (30), which can be a potential mode to complement glycolysis. However, this theory is still to be extensively established. A more common and well-established finding is that in hypoxia, cancer cells shift their metabolism toward lactic acid fermentation in order to produce energy while bypassing the tricarboxylic acid cycle and oxidative phosphorylation (31).

The altered metabolic scenario in hypoxic tumor cells remains to be unraveled. Moreover, the epigenetic regulatory mechanism behind this altered pathway is of crucial importance and must be delineated as well. In this scenario, the present study aims to elucidate the metabolic regulations in hypoxic tumor cells, particularly in the context of glucose metabolism, from an epigenetic perspective. The epigenetic regulator ZMYND8 is found to be induced at both the RNA and protein levels upon induction of hypoxia in 2D and 3D cell culture systems, as well as in breast cancer patients, showing a positive correlation with the hypoxia marker. Subsequently, a positive correlation is observed between ZMYND8 and the glucose metabolic genes, including *HKII* and lactate dehydrogenase A (*LDHA*). Metabolic flux measurements show that ZMYND8 significantly suppresses glycolysis in the hypoxic condition, leading to an activation of the lactate pools in the cells. Overall, the present study elucidates a novel role of ZMYND8 in regulating carbohydrate metabolic programs under hypoxic conditions, which directly impacts tumorigenicity.

## Results

### Epigenetic regulator ZMYND8 expression is augmented upon hypoxia induction

**Figure 1:**
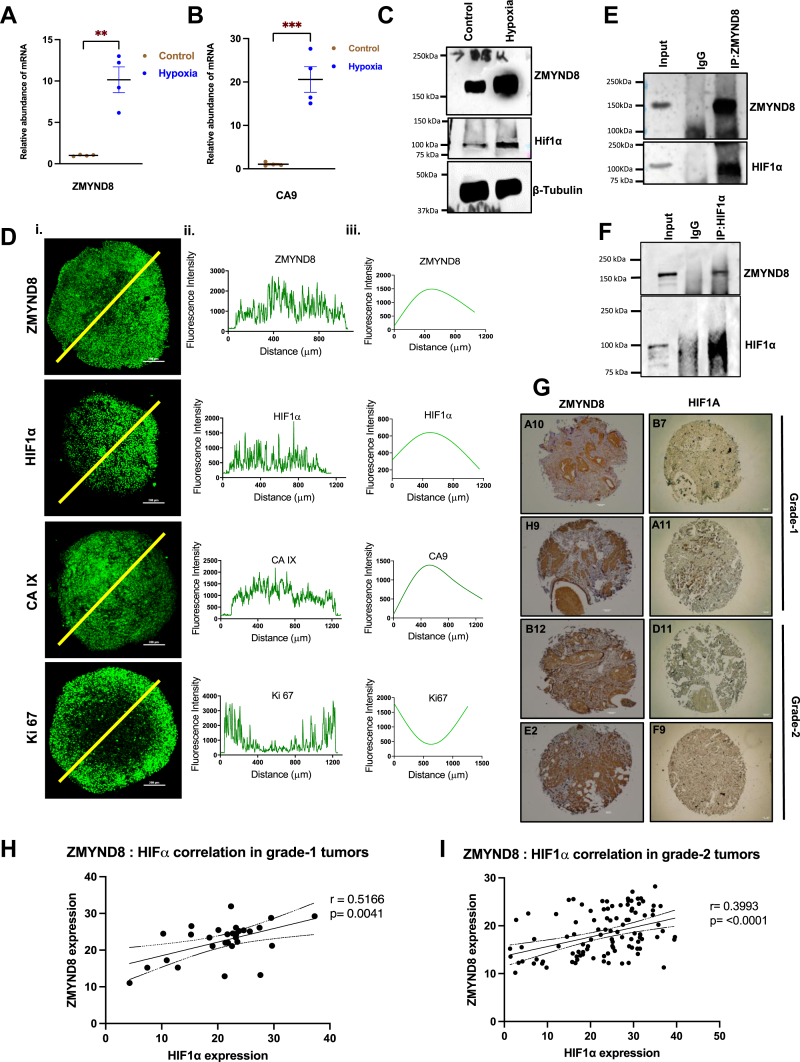
**ZMYND8 expression increases upon induction of hypoxia.***A* – *B*, qRT PCR analysis of *ZMYND8* and *CAIX* expression under hypoxic condition (1% O_2_). An unpaired *t* test was performed to analyze the *p*-value significance (∗*p* < 0.05; ∗∗*p* < 0.01; ∗∗∗*p* < 0.001; ns, nonsignificant (*p* > 0.05)) for the statistical analyses, the error bar represents the standard error of the mean (SEM). *C*, western blot image showing the protein level expression of ZMYND8 and HIF⍺ in hypoxic conditions. *D*, immunofluorescence imaging (i), fluorescence intensity profile (ii) and “Fit Spline” representation (iii) of MCTS showing expression distribution of ZMYND8, HIF1⍺, CAIX, and Ki-67 as a function of the indicated distance. *E* – *F*, coimmunoprecipitation shows the interaction of endogenous HIF1A and ZMYND8 in hypoxia-treated MCF7 cells. *G*, immunohistochemistry of ZMYND8 and HIF1α from tumor microarray. *H* – *I*, correlation plot of ZMYND8 and HIF1α absolute expression values. Pearson correlation analysis was performed to calculate the *p*-value significance, and simple linear regression analysis was done to calculate the R value. ZMYND8, zinc finger MYND-type containing 8; HIF1⍺, hypoxia-inducible factor 1-⍺; MCTS, multicellular tumor spheroids; CAIX, carbonic anhydrase IX.

To investigate the relationship between hypoxia and ZMYND8 expression, we exposed cancer cells to hypoxic conditions. Bearing in mind that the dynamic functional regulation of ZMYND8 has been explored in quite in-depth in triple negative breast cancer (32, 33) and HER2-positive breast cancer (34), but fewer mechanistic studies are available on luminal breast cancer, which is of a more frequent occurrence, we initiated our studies in the luminal breast cancer cell line T47D. Quantitative real-time PCR (qRT-PCR) analysis revealed a significant increase in mRNA levels of *ZMYND8* and *CAIX* under hypoxic conditions compared to normoxic controls in T47D (Fig. 1, *A* and *B*). Western blot analysis further confirmed the upregulation of ZMYND8 at the protein level under hypoxic conditions (Fig. 1*C*). Concurrently, we observed stabilization of HIF1α, a key regulator of cellular response to hypoxia, under hypoxic conditions (Fig. 1*C*).

Multicellular tumor spheroids (MCTS) are aggregation-based 3D structures formed by the cancer cells without the assistance of any scaffold. They mimic certain tumor features, such as heterogeneous cell populations, drug resistance, and elicit gradients of oxygen across the dimension of tumor spheroids (35). Here, we used T47D-based tumor spheroids and performed immunofluorescence staining with different markers. Ki-67 marked the proliferative tumor peripheral region and was found to be appreciably reduced in the MCTS central region (Figs. 1*D*, S1). On the other hand, staining with anti-carbonic anhydrase IX (CAIX) antibody showed a greater distribution of CAIX in the central region compared to the periphery (Figs. 1*D*, S1). CAIX is a hypoxia target enzyme which is regulated by HIF1α (36). Immunofluorescence of HIF1α showed distribution mainly in the hypoxic central portions of the MCTS (Figs. 1*D*, S1). Remarkably, ZMYND8 also elicited differential expression within the spheroid structure, with higher expression in the hypoxic central region compared to the periphery (Figs. 1*D*, S1). The fluorescent intensity measurement of Ki-67, CAIX, HIF1α, and ZMYND8 across demarcated diameter established their differential distribution in the MCTS (Fig. S1).

To explore potential interactions between ZMYND8 and HIF1α, we performed coimmunoprecipitation experiments using an anti-ZMYND8 antibody from luminal breast cancer cells, MCF7, exposed to hypoxia. Our results revealed that endogenous ZMYND8 and HIF1α physically interact under hypoxic conditions (Fig. 1*E*), suggesting a possible functional relationship between these two proteins in the cellular response to hypoxia. Furthermore, reverse coimmunoprecipitation experiments with anti-HIF1α antibody also confirmed the interaction between ZMYND8 and HIF1α in MCF7 cells under hypoxia (Fig. 1*F*).

To investigate the relationship between ZMYND8 and HIF1α expression in luminal breast cancer specimens, we performed immunohistochemistry (IHC) on the tumor microarray. The IHC analysis revealed variable expression patterns of ZMYND8 and HIF1α across the tumor samples, which were subsequently quantified. Notably, a correlation analysis using the absolute expression values obtained from the IHC data revealed a strong positive correlation between ZMYND8 and HIF1α expression levels in grade 1 (r = 0.543, *p* = 0.0023) and grade 2 tumors (r = 0.3993, p = <0.0001) (Fig. 1, *G*–*I*). We subsequently analyzed the expression of ZMYND8 and HIF1α in parallel sections of the same luminal breast cancer specimen and quantified their expression, calculating correlations across multiple imaging fields. In four such breast cancer specimens, a significant correlation was observed between the two proteins (Fig. S2).

These findings collectively demonstrate that ZMYND8 expression is upregulated under hypoxic conditions in breast cancer cells, both at the mRNA and protein levels, and that ZMYND8 interacts with HIF1α as part of the cellular response to hypoxia. Further, our results suggest that the relationship between ZMYND8 and HIF1α may have clinical relevance in breast cancer, particularly in hormone receptor-positive subtypes.

### ZMYND8 is associated with the activation of carbohydrate metabolic genes during hypoxia

**Figure 2:**
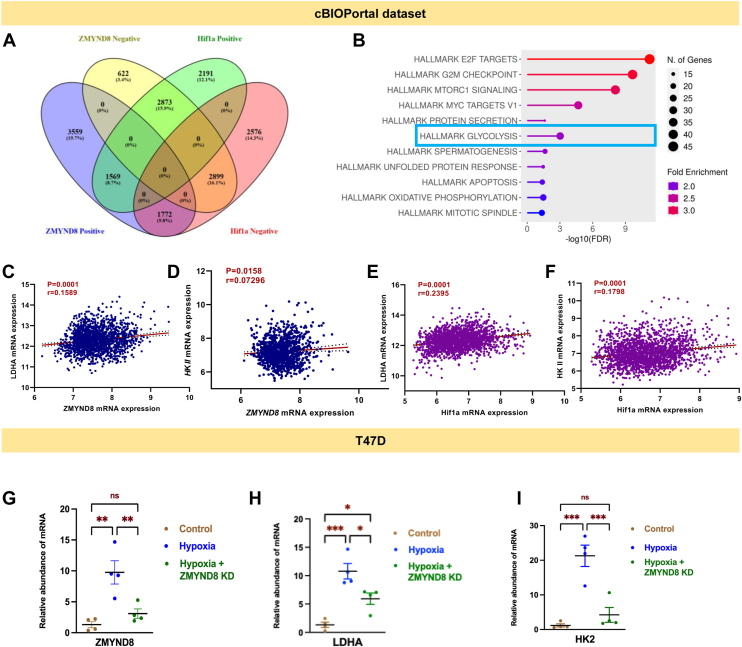
**ZMYND8 regulates a wide array of carbohydrate metabolic genes inERandPR-positive breast tumors**. *A*, venn diagram analysis of ZMYND8 correlated genes from cBioPortal database. *B*, the hallmark DB analysis was performed by using the ShinyGO webserver. *C*–*F*, correlation plot of *ZMYND8-HKII*, *ZMYND8-LDHA* and *HIF1⍺-HKII* and *HIF1⍺-LDHA* expression levels from cBioPortal database. Pearson correlation analysis was performed to calculate the *p*-value significance and simple linear regression analysis was done to calculate the R value. *G*–*I*, qRT-PCR analysis from T47D cells showing *ZMYND8*, *LDHA*, and *HKII* expression under control, hypoxia, and *ZMYND8* knock down in hypoxic condition. One-way ANOVA was performed to analyze the *p*-value significance (∗*p* < 0.05; ∗∗*p* < 0.01; ∗∗∗*p* < 0.001; ns, nonsignificant (*p* > 0.05)) for the statistical analyses, the error bar represents the standard error of the mean (SEM). ER, estrogen receptor; LDHA, lactate dehydrogenase A; ZMYND8, zinc finger MYND-type containing 8; HIF1⍺, hypoxia-inducible factor 1-⍺; PR, progesterone receptor.

To elucidate the potential role of ZMYND8 in breast cancer metabolism, we analyzed *ZMYND8*-correlated genes from expression data (37, 38) in the cBioPortal database. Venn diagram analysis shows 1569 candidate genes showing positive expression of *ZMYND8* (Fig. 2*A*). Using the ShinyGO webserver, which revealed significant enrichment in metabolic pathways in terms of the number of genes as well as its fold enrichment score (Fig. S3*A*). This finding suggests a potential link between ZMYND8 and the regulation of cellular metabolism in breast cancer. Significant biological pathway analysis of *ZMYND8*-correlated genes found under the “metabolic pathways” hub, executed on the ShinyGo webserver, identified several metabolic gene hubs, including glycolysis, gluconeogenesis, and oxidative phosphorylation (Fig. S3*C*). Analysis of the *ZMYND8* correlated metabolic gene hub also demonstrated high enrichment of the HIF1α signaling pathway, indicating an association between ZMYND8 and HIF1α in regulating breast cancer metabolism (Fig. S3*B*). Furthermore, Hallmark gene signature analysis from the ShinyGo database demonstrated the presence of glycolysis to be enriched from ZMYND8 correlated genes, further strengthening the ZMYND8-HIF1α correlation in regulating the metabolism in breast cancer (Fig. 2*B*). These results indicate that ZMYND8 may play a role in transcriptional regulation of metabolic genes in breast cancer.

To further investigate the relationship between ZMYND8 and specific metabolic genes, we performed correlation analyses using expression data from the cBioPortal database (37, 38). Our analysis revealed significant positive correlations between *ZMYND8* expression and key glycolytic genes, including *HKII* and *LDHA* (Fig. 2, *C* and *D*). Further, correlation analysis of *HIF1α* and *LDHA* and *HKII* mRNA expression showed positive correlation, further associating ZMYND8-mediated regulation of breast cancer metabolism with HIF1α (Fig. 2, *E* and *F*). To investigate the role of ZMYND8 in regulating metabolic genes under hypoxia, we examined the expression of ZMYND8 and key metabolic enzymes, HKII and LDHA, under normoxic and hypoxic conditions in luminal breast cancer cells T47D. Furthermore, we knocked down *ZMYND8* under hypoxia to monitor the contribution of ZMYND8 in regulating metabolic gene expression. A significant increase in *ZMYND8* mRNA levels under hypoxic conditions compared to normoxia could be observed (Fig. 2*G*). Notably, the expression of *LDHA* and *HKII* also increased significantly under hypoxia and decreased significantly upon *ZMYND8* knockdown (Fig. 2, *G*–*I*).

**Figure 3:**
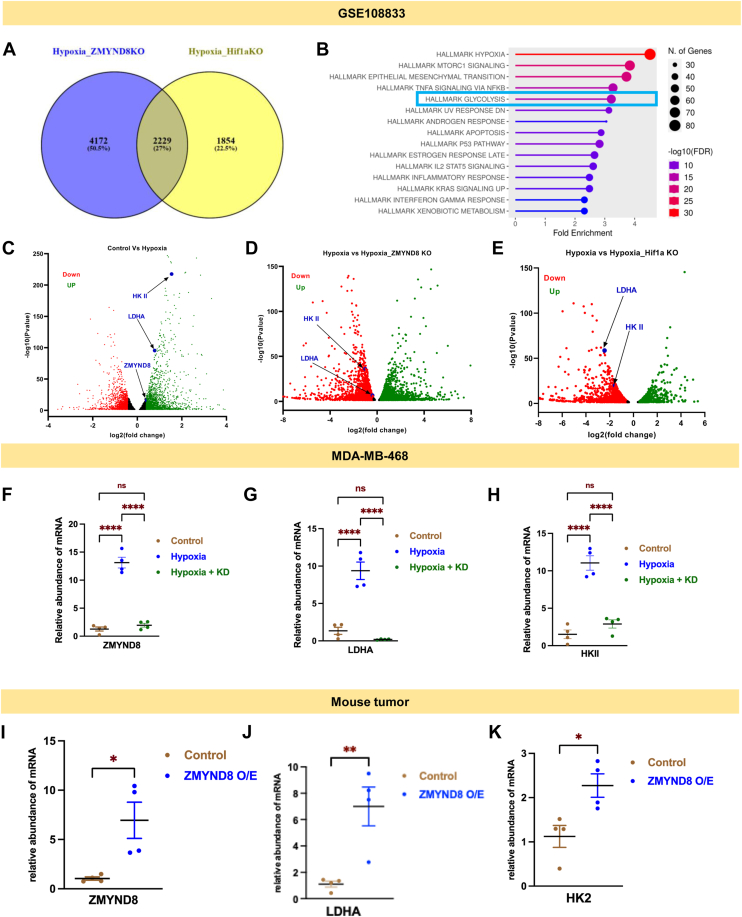
**ZMYND8/HIF1a transcriptionally regulate the *LDHA* gene transcription**. *A*, venn diagram analysis of MDA-MB-231 cells from the DEGs between hypoxic and *ZMYND8* knockout in hypoxia, and hypoxia and HIF1⍺ KO in hypoxic conditions. *B*, the hallmark DB analysis was performed by using the ShinyGO webserver. *C*–*E*, volcano plot analysis with log_2_(FC) in control and hypoxia and *ZMYND8* and *HIF1⍺* KO in hypoxic conditions from the DEGs show expression of *LDHA* and *ZMYND8* and *HKII*. *F*–*H*, qRT-PCR assay from MDA-MB-468 cells showing *ZMYND8*, *LDHA* and *HKII* expression under control, hypoxia, and ZMYND8 knockdown in hypoxic condition. *I*–*K*, qRT-PCR assay from mouse tumor showing expression of *ZMYND8*, *LDHA*, and *HKII* respectively, grafted with control and *ZMYND8* overexpressed 4T1 cells. One-way ANOVA was performed to analyze the *p*-value significance (∗*p* < 0.05; ∗∗*p* < 0.01; ∗∗∗*p* < 0.001; ns, nonsignificant (*p* > 0.05)) for the statistical analyses, the error bar represents the standard error of the mean (SEM). LDHA, lactate dehydrogenase A; ZMYND8, zinc finger MYND-type containing 8; HIF1⍺, hypoxia-inducible factor 1-⍺; DEG, differentially expressed gene.

**Figure 4:**
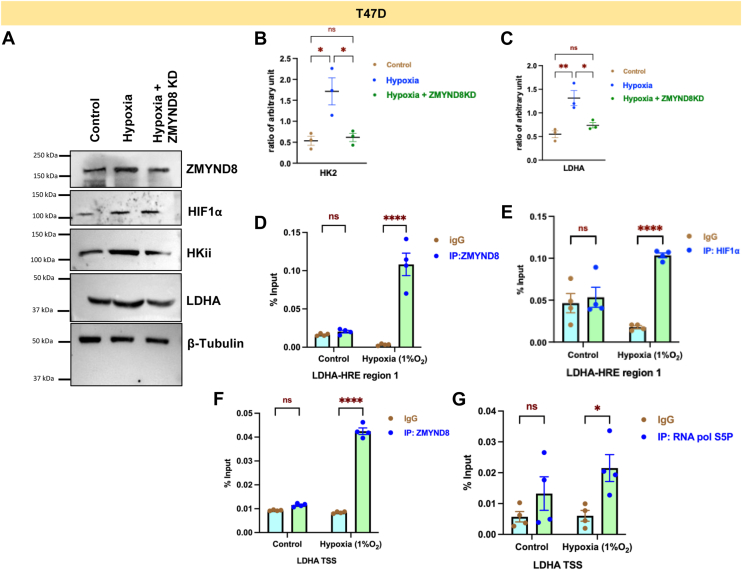
**ZMYND8/HIF1α corecruitment regulates LDHA transcription.***A*, western blot images showing expression of LDHA, ZMYND8 and HIF1⍺ in T47D cells. *B* – *C*, scattered plots showing normalized densitometric quantification of western blot band intensities. One-way ANOVA was performed to analyze the *p*-value significance (∗*p* < 0.05; ∗∗*p* < 0.01; ∗∗∗*p* < 0.001; ns, nonsignificant (*p* > 0.05) for the statistical analyses. The error bar represents the standard error of the mean (SEM). *D*, ChIP-qPCR assays showing recruitment of ZMYND8 in HRE element of *LDHA* promoter in control and hypoxic conditions. *E*, ChIP-qPCR assays showing recruitment of HIF1⍺ in HRE of *LDHA* promoter in control and hypoxic conditions. *F*, ChIP-qPCR assays showing recruitment of ZMYND8 around TSS of *LDHA* promoter in control and hypoxic conditions. *G*, ChIP-qPCR assays showing recruitment of RNA pol S5P in HRE element of *LDHA* promoter in control and hypoxic conditions. Two-way ANOVA was performed to analyze the *p*-value significance (∗*p* < 0.05; ∗∗*p* < 0.01; ∗∗∗*p* < 0.001; ns, nonsignificant (*p* > 0.05)) for the statistical analyses. The error bar represents the standard error of the mean (SEM). LDHA, lactate dehydrogenase A; ZMYND8, zinc finger MYND-type containing 8; HIF1⍺, hypoxia-inducible factor 1-⍺; HRE, hypoxia response element; TSS, transcriptional start site.

To delve into the role of ZMYND8-mediated regulation of metabolic genes beyond luminal breast cancer subtype, we have reanalyzed a published RNA sequencing dataset from the GEO database (GSE108833). Venn diagram analysis showed expression of 2229 genes being perturbed upon *ZMYND8* or *HIF1α* knockout (Fig. 3*A*). Hallmark DB analysis from ShinyGo webserver showed hallmark gene hubs related to hypoxia and glycolysis (Fig. 3*B*). Volcano plot from the differentially expressed genes in control and hypoxia in MDA-MB-231 cells showed *ZMYND8*, *LDHA*, and *HKII* mRNA levels were upregulated in hypoxic conditions (Fig. 3*C*). Remarkably, *ZMYND8/HIF1α* knockout in hypoxic conditions showed downregulation of *LDHA* and *HKII* potentially implying direct role of ZMYND8 and HIF1α in transcriptional regulation of these genes (Fig. 3, *D* and *E*). Validation experiments in MDA-MB-468 cells unraveled that *ZMYND8* knockdown in hypoxic conditions downregulates mRNA expression of *HKII* and *LDHA* genes, advocating the role of ZMYND8-mediated regulation of essential carbohydrate metabolic genes beyond luminal breast cancer subtype (Fig. 3, *F*–*H*). Experiments in HEK 293T cells also showed significant downregulation of *HKII* and *LDHA* upon *ZMYND8* knockdown in hypoxic conditions, indicating the tissue-type independent nature of ZMYND8 in regulating metabolic genes like *LDHA* and *HKII* (Fig. S4, *A*–*C*). An mRNA stability assay performed in control and hypoxic conditions, with or without *ZMYND8*, indicates no significant change in mRNA stability, implying that changes in mRNA abundance are due to transcriptional regulation (Fig. S4, *D* and *E*). Western blot analysis corroborated these findings at the protein level. Hypoxia induced the expression of HIF1α, ZMYND8, LDHA, and HKII in luminal breast cancer cells, T47D (Figs. 4, *A*–*C*, S5*B*). *ZMYND8* knockdown under hypoxic conditions resulted in decreased protein levels of LDHA and HKII, while HIF1α levels remained elevated. Moreover, western blot analysis from MDA-MB-468 cells also showed a marked decrease in HKII and LDHA expression, indicating the subtype-independent nature of regulation mediated by ZMYND8 in breast cancer (Fig. S5*A*). Western blot analysis from HEK 293T cells follows a similar pattern, indicating the tissue-type independent nature of transcriptional regulation of LDHA and HKII by ZMYND8 (Fig. S5*C*). Remarkably, qRT PCR from harvested tumors from BALB/c mice bearing WT 4T1 or *ZMYND8* overexpressed 4T1 cells showed a significant increase in *LDHA* and *HKII* expression, indicating the regulation of metabolic genes by ZMYND8 in the *in vivo* context (Fig. 3, *I* and *J*).

To explore the mechanism by which ZMYND8 regulates metabolic genes, we performed chromatin immuno precipitation-quantitative real-time PCR (ChIP-qPCR) experiments focusing on the *LDHA* promoter at the HRE sites. The results showed significant enrichment of ZMYND8 along with HIF1α at the *LDHA* promoter compared to the Immunoglobulin G control, confirming the direct binding of ZMYND8/HIF1α complex to the *LDHA* gene regulatory region (*LDHA*-HRE region 1 and 2) (Figs. 4, *D* and *E*, S6, *A* and *B*). We subsequently performed ChIP-qPCR for ZMYND8 along with RNA polymerase II at the transcriptional start site (TSS) of the *LDHA* gene (*LDHA* TSS) (Fig. 3, *F* and *G*). ChIP-qPCR assay of HIF1α and ZMYND8 performed on the gene body region, taken as negative control (*LDHA* intergenic region; -ve control), indicates no significant recruitment of ZMYND8 and HIF1α (Fig. S6, *A* and *B*), pointing toward the specificity of the assay and selective recruitment of both factors on chromatin.

These findings suggest that ZMYND8 may have specific and differential associations with various metabolic genes in breast cancer. Further, it emerges that ZMYND8 plays a crucial role in the transcriptional regulation of key metabolic genes, particularly *LDHA* and *HKII*, under hypoxic conditions in breast cancer cells. The data suggest a mechanism whereby ZMYND8 cooperates with HIF1α to modulate the expression of these metabolic genes, potentially contributing to the metabolic adaptations of cancer cells in hypoxic environments.

### ZMYND8 promotes glycolysis and lactate production under hypoxic conditions

**Figure 5:**
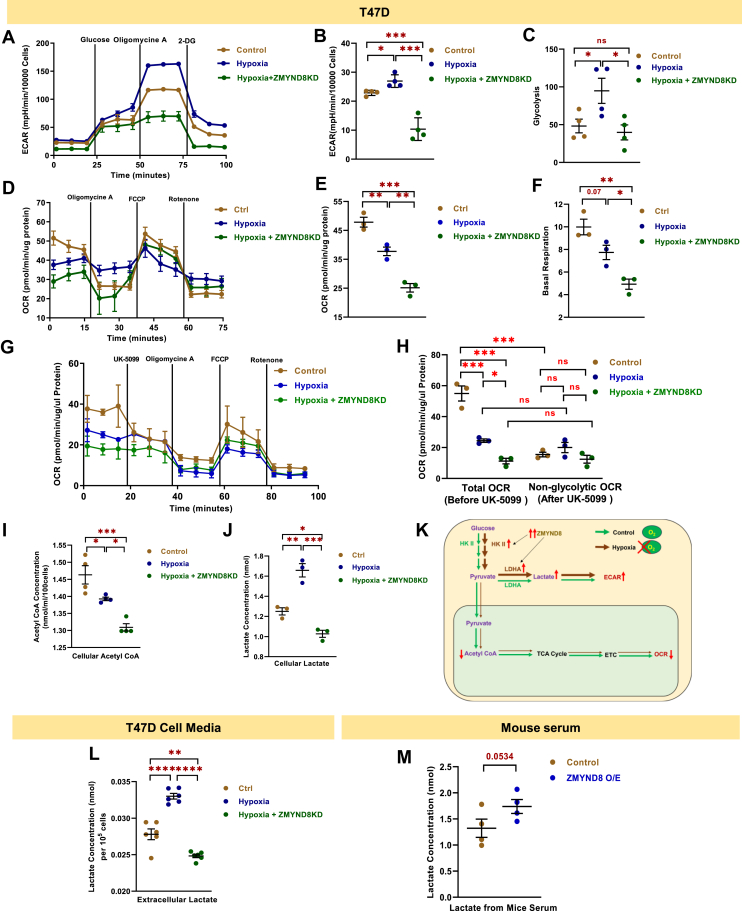
**ZMYND8 suppresses immune invasion by promoting lactate production and extracellular acidosis.***A*, measurement of extracellular acidification rate using Seahorse flux analyzer in control, hypoxia, and ZMYND8 knockdown in hypoxic condition. *B*, quantification of extracellular acidification rate using Seahorse flux analyzer in control, hypoxia, and *ZMYND8* knockdown in hypoxic condition. *C*, quantification of glycolysis measured *via* Seahorse flux analyzer in control, hypoxia, and *ZMYND8* knockdown in hypoxic condition in T47D cells. *D*, measurement of oxygen consumption rate using Seahorse flux analyzer in control, hypoxia, and *ZMYND8* knockdown in hypoxic condition. *E* – *F*, quantification of oxygen consumption rate and basal respiration using Seahorse flux analyzer in control, hypoxia, and *ZMYND8* knockdown in hypoxic condition. *G*, measurement of UK-5099 dependent oxygen consumption rate using Seahorse flux analyzer in control, hypoxia, and *ZMYND8* knockdown in hypoxic condition. *H*, quantification of oxygen consumption rate before and after UK-5099 treatment in control, hypoxia, and *ZMYND8* knockdown in hypoxic conditions. *I* – *J*, measurement of cellular acetyl-CoA and lactate concentration in T47D cells in control, hypoxia, and *ZMYND8* knockdown in hypoxic conditions. *K*, graphical representation of ZMYND8-mediated regulation of cellular metabolic flux in control and hypoxic conditions. *L*, measurement of extracellular lactate concentration in control, hypoxia, and *ZMYND8* knockdown in hypoxic conditions. One-way ANOVA was performed to analyze the *p*-value significance (∗*p* < 0.05; ∗∗*p* < 0.01; ∗∗∗*p* < 0.001; ns, nonsignificant (*p* > 0.05)) for the statistical analyses, the error bar represents the standard error of the mean (SEM). *M*, measurement of serum lactate concentration from animals injected with control 4T1 cells and *ZMYND8* overexpressed 4T1 cells. An unpaired *t* test was performed to analyze the *p*-value significance (∗*p* < 0.05; ∗∗*p* < 0.01; ∗∗∗*p* < 0.001; ns, nonsignificant (*p* > 0.05)) for the statistical analyses. The error bar represents the standard error of the mean (SEM). ZMYND8, zinc finger MYND-type containing 8

To investigate the functional impact of ZMYND8 on cellular metabolism under hypoxic conditions, we performed a series of metabolic assays in T47D cells. Extracellular acidification rate (ECAR) measurements revealed a significant increase in ECAR and glycolytic activity under hypoxia compared to normoxia (Figs. 5, *A*–*C*, S7, *A* and *B*). Notably, *ZMYND8* knockdown in hypoxic conditions significantly reduced this hypoxia-induced increase in ECAR (Fig. 5, *A* and *B*). Further analysis of glycolytic parameters revealed that hypoxia significantly enhanced glycolysis and glycolytic capacity compared to normoxic conditions. *ZMYND8* knockdown under hypoxia partially reversed these effects, considerably reducing both glycolysis and glycolytic capacity (Figs. 5*C*, S7*A*. Nonglycolytic acidification was also increased under hypoxia and reduced upon *ZMYND8* knockdown (Fig. S7*B*).

Oxygen consumption rate (OCR) measurements showed a significant decrease under hypoxic conditions compared to normoxia (Fig. 5, *D*–*F*). Interestingly, *ZMYND8* knockdown under hypoxia further reduced OCR (Fig. 5, *D*–*F*). Basal respiration followed a similar trend, with a significant decrease observed in hypoxia and a further reduction upon *ZMYND8* knockdown (Fig. 5*F*). To determine the contribution of ZMYND8 in regulating the glucose metabolism leading to alteration in OCR during hypoxia, cells were treated with UK-5099 to block pyruvate transport to mitochondria (Figs. 5, *G* and *H*, S7*C*). UK-5099 treatment decreased OCR in control cells but had minimal effect on hypoxic cells (Fig. 5*H*). Quantification of UK-5099-responsive OCR showed significant reduction under hypoxic conditions, with no additional change upon *ZMYND8* knockdown (Fig. S7*C*). Remarkably, the total OCR decreased significantly under hypoxia and was further reduced by *ZMYND8* knockdown (Fig. 5*H*). In contrast, nonglycolytic OCR (OCR remaining after UK-5099 treatment) remained unchanged across hypoxic and *ZMYND8* knockdown conditions (Fig. 5*H*). To assess the metabolic consequences of these changes instilled by ZMYND8, cellular acetyl-CoA levels were measured, which showed a significant decrease upon hypoxia induction, and were further reduced upon *ZMYND8* knockdown (Fig. 5*I*). The reduced cellular acetyl-CoA pools reflected an overall reduction in glycolysis (Fig. 5, *A*–*C*, and *K*) (14).

To validate the role of ZMYND8 in anaerobic lactate fermentation and extracellular acidosis, we measure the cellular and extracellular lactate pool upon both hypoxia and *ZMYND8* knockdown during hypoxic conditions. Interestingly, we found both cellular and extracellular lactate amounts increased during hypoxia and decreased dramatically upon knocking down *ZMYND8* (Fig. 5, *J* and *L*). Remarkably, measurement of serum lactate collected from BALB/c mice bearing tumors with control 4T1 or *ZMYND8* overexpressed 4T1 cells showed a significant increase in lactate concentration in the latter scenario (Fig. 5*M*). This indicates that the regulation of LDHA by ZMYND8 encompasses not only 2D and 3D cell culture models but also the *in vivo* context (Fig. 5*M*).

These results collectively demonstrate that ZMYND8 plays a crucial role in modulating carbohydrate metabolism under hypoxic conditions in breast cancer cells. Further, ZMYND8 promotes glycolysis and lactate production while suppressing oxidative phosphorylation in response to hypoxia (Fig. S7*D*). The attenuation of these metabolic changes upon *ZMYND8* knockdown suggests that ZMYND8 is a key mediator of the metabolic adaptations to hypoxia in these cancer cells.

### ZMYND8 suppresses immune cell invasion in the hypoxic tumor microenvironment

**Figure 6:**
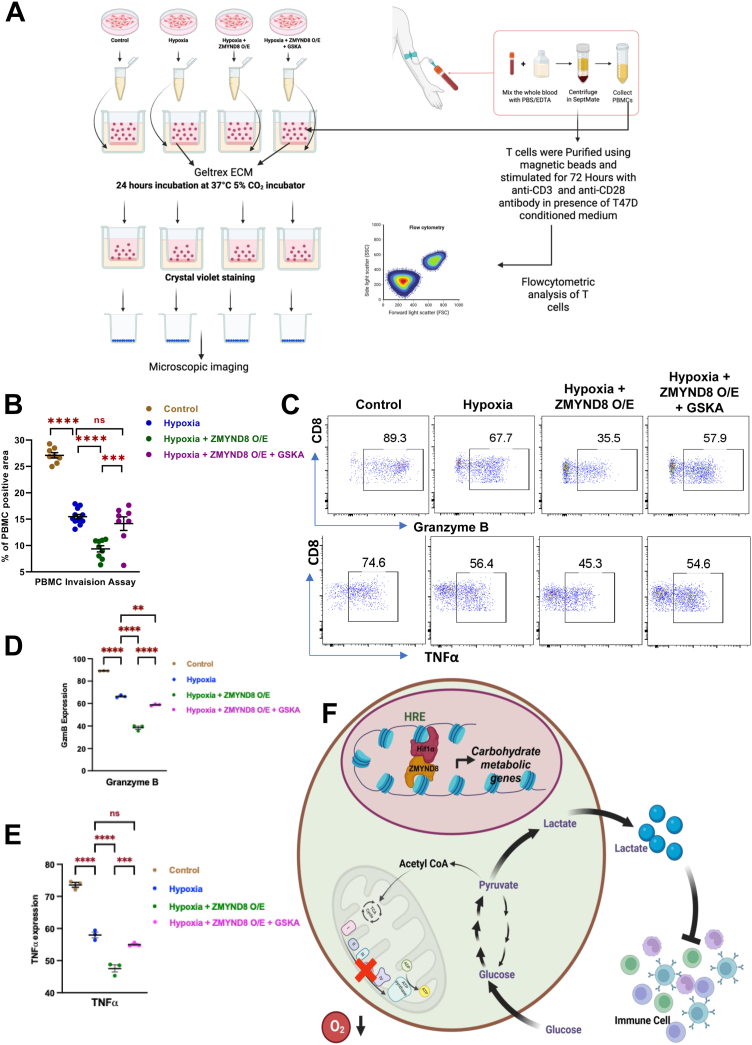
**ZMYND8 regulates the tumor microenvironment**. *A*, graphical representation of immune invasion assay. *B*, quantification of invaded PBMC through the matrix in the presence of conditioned medium from cells cultured in control, hypoxia, and *ZMYND8* overexpression in hypoxic condition in the presence and absence of GSKA. One-way ANOVA was performed to analyze the *p*-value significance (∗*p* < 0.05; ∗∗*p* < 0.01; ∗∗∗*p* < 0.001; ns, nonsignificant (*p* > 0.05)) for the statistical analyses, the error bar represents the standard error of the mean (SEM). *C*–*E*, flow cytometry image and quantification showing percentage of granzyme B and TNFα positive cells in control, hypoxia, and *ZMYND8* overexpression in hypoxic condition in presence and absence of GSKA. One-way ANOVA was performed to analyze the *p*-value significance (∗*p* < 0.05; ∗∗*p* < 0.01; ∗∗∗*p* < 0.001; ns, nonsignificant (*p* > 0.05)) for the statistical analyses, the error bar represents the standard error of the mean (SEM). *F*, graphical representation showing the function of ZMYND8 in elevated lactate production and the effect of secretory lactate in supressing immune cell activity. ZMYND8, zinc finger MYND-type containing 8; TNFα, tumor necrosis factor α; PBMC, peripheral blood mononuclear cell.

Extracellular lactate is well known to suppress immune invasion and tumor progression (39). To investigate the role of extracellular lactate in immune suppression, we conducted a peripheral blood mononuclear cell (PBMC) invasion assay, which depicts the effect of cancer cell secretome on the PBMCs. In the presence of the conditioned medium from the cancer cells, there is an alteration in the chemotaxis ability of the PBMCs, which mimics how the immune cells are being called up in the tumor microenvironment depending upon the cytokine gradients. In order to delineate the role of ZMYND8 on immune cell invasion, the conditioned medium from cells cultured under four distinct conditions-control, hypoxia, and *ZMYND8* overexpression in hypoxic conditions, in the absence or presence of LDHA noncompetitive inhibitor (GSK2837808A; 100 nM) added to the PBMC to monitor their invasion property (Figs. 6, *A* and *B*, S8*A*). Bright field microscopy images following crystal violet staining revealed differential PBMC migration through the matrix across the four experimental conditions (Figs. 6*B*, S8*A*). Quantitative analysis of invading PBMCs, provided compelling evidence for the regulatory role of ZMYND8 in immune cell invasion (Fig. 6*B*). Notably, a significant decrease in PBMC invasion under hypoxic conditions compared to normoxia was observed, which further decreased upon over expressing *ZMYND8* (Figs. S8*A*, 6*B*). Remarkably, employing LDHA noncompetitive (GSK2837808A; 100 nM) inhibitors led to a rescue of the PBMC invasion (Figs. S8*A*, 6*B*) (40).

Circulatory lactate is an important player in regulating T cell response in murine models (41). The role of extracellular lactate in conditioned medium of T47D cells under control, hypoxia, and *ZMYND8* over expression in hypoxic conditions was next investigated in CD8+ T cells (Fig. 6*A*). The results indicate a dramatic drop in a very crucial effector CD8+ T cell cytokine (42), granzyme B in hypoxic conditions compared to the control condition (Fig. 6, *C* and *D*). Interestingly, *ZMYND8* over expression in hypoxic conditions led to decrease the expression of granzyme B further (Fig. 6, *C* and *D*), and this effect was rescued by employing noncompetitive inhibition of LDHA (Fig. 6, *C* and *D*). Moreover, another crucial T cell cytokine, tumor necrosis factor α (TNFα) which plays important role in naive and effector T cell proliferation (43), showed similar effects in control, hypoxia, and *ZMYND8* over expression in the presence or absence of noncompetitive inhibitor of LDHA (Fig. 6, *C* and *E*). Differentiation and augmentation of CD8+ T cell activation demarcated by expression of class II MHC molecules on CD8+ T cells rely heavily on interferon γ (IFNɣ) (44), which also showed a similar trend in control, hypoxia, *ZMYND8* overexpression and *ZMYND8* overexpression in presence or absence of noncompetitive inhibition of LDHA under hypoxic conditions.

These findings suggest that ZMYND8 plays a crucial role in promoting immune cell evasion under hypoxic conditions, a common feature of the tumor microenvironment. Based on our results, we propose a mechanism by which ZMYND8 regulates lactate pools within cells, subsequently influencing immune cell invasion (Fig. 6*H*). This model provides a potential explanation for the relay of metabolic flux toward increased lactate production during hypoxia, as fine-tuned in the context of gene expression by the ZMYND8 and HIF1α complex. The extracellular lactate plays a crucial role in suppressing PBMC invasion, underscoring the importance of ZMYND8 in modulating the tumor microenvironment (Fig. 6*H*).

Taken together, these results indicate that under hypoxic conditions, ZMYND8 promotes anaerobic glucose metabolism, leading to excess lactate production and extracellular acidosis, which in turn causes immune suppression. Remarkably, ZMYND8 is a key regulator of immune cell invasion in the context of tumor hypoxia, offering new insights into the complex interactions within the tumor microenvironment.

## Discussion

The role of epigenetics in cellular response to hypoxia has been an area of intense research in recent years. Hypoxia induces global changes in the epigenetic landscape, including alterations in DNA methylation patterns, histone modifications, and chromatin remodeling (45, 46). These changes are crucial for the transcriptional reprogramming that enables cells to adapt to low-oxygen environments. For instance, hypoxia has been shown to induce global DNA hypomethylation while causing site-specific hypermethylation of certain gene promoters (47). Histone modifications, particularly H3K4 methylation and H3K27 acetylation, have been implicated in the activation of hypoxia-responsive genes (48). Moreover, histone demethylases such as KDM3A and KDM4B have been identified as direct HIF target genes (49, 50), further emphasizing the intricate relationship between hypoxia and epigenetic regulation.

In this context, our study introduces ZMYND8 as a novel epigenetic player in the hypoxic response of breast cancer cells. We provide compelling evidence for the critical role of ZMYND8 in breast cancer metabolism, particularly under hypoxic conditions. The elevated expression of ZMYND8 under hypoxia, and its association with HIF-1α, both *in vitro* and in patient samples, suggests a conserved mechanism essential for transcriptional adaptation to hypoxic stress.

Our analysis of ZMYND8-correlated patient transcriptome revealed a significant impact on metabolic pathways, particularly glycolysis. The positive correlation between ZMYND8 expression and key glycolytic enzyme, HKII, and anaerobic respiratory enzyme LDHA, underscores its importance in metabolic reprogramming. This is further supported by ChIP assay results, demonstrating ZMYND8 recruitment to the *LDHA* gene promoter. Functional consequences of ZMYND8-mediated transcriptional regulation were evident in metabolic flux analyses, showing an increase in ECAR and a decrease in OCR under hypoxia. Interestingly, while increased ECAR levels could be reversed by *ZMYND8* knockdown, OCR levels showed a further drop, indicating that ZMYND8 primarily programs glycolytic pathways rather than OXPHOS in a hypoxic environment.

Interestingly, the differential response to UK-5099 provides mechanistic insights into how ZMYND8 influences glucose-dependent OCR levels. Under normoxic conditions, pyruvate transport blockade significantly reduced OCR, indicating robust glucose-dependent mitochondrial respiration. However, under hypoxia, a metabolic shift toward anaerobic fermentation could be clearly observed. The inability of *ZMYND8* knockdown to further alter UK-5099-responsive OCR under hypoxia suggests that the primary role of ZMYND8 is not in directly regulating pyruvate transport, but rather in controlling the overall flux through glycolysis that determines substrate availability for mitochondrial metabolism. Our model suggests that ZMYND8 functions as a metabolic switch that, when activated by hypoxia, promotes glucose flux through anaerobic fermentation while simultaneously maintaining sufficient glycolytic capacity to support cellular energy demands. The bifurcation of glucose metabolism toward lactate production, as evidenced by increased extracellular acidification, represents an adaptive response that prioritizes rapid ATP generation over metabolic efficiency.

The connection between metabolic reprogramming and immune signaling in breast cancer is a particularly intriguing aspect of our findings. The observed reduction in PBMC invasion in response to conditioned media from hypoxic cells, which was dampened upon *ZMYND8* overexpression, suggests that the lactate-rich, acidic microenvironment created by ZMYND8 activity may contribute to tumor immune evasion. Recent studies have shown that increased lactate production by tumor cells can have immunosuppressive effects, including inhibition of T cell proliferation and function (41, 51), as well as promoting the differentiation of tumor-associated macrophages toward an M2-like phenotype (52). Our results suggest that ZMYND8 may be a key player in this process, linking epigenetic regulation, metabolic reprogramming, and immune evasion in breast cancer. Furthermore, our results involving CD8+ T cells indicate that ZMYND8-mediated sustained production of lactate directly affects T cell functionality, as noted in a dramatic drop in the expression of granzyme B, tumor necrosis factor α, and interferon γ (IFNγ) in hypoxia compared to normoxia, and further reduction in the overexpression of ZMYND8, with concomitant rescue under presence of LDHA inhibitor.

The epigenetic landscape of cancer cells is known to be dynamic and responsive to microenvironmental cues. The upregulation of ZMYND8 under hypoxic conditions and its subsequent impact on metabolic gene expression represent an important example of how the tumor microenvironment can shape the epigenetic state of cancer cells, influencing both the metabolic phenotype of the tumor and its interactions with the immune system.

Our findings of decreased acetyl-CoA levels and increased lactate production in the context of ZMYND8-mediated metabolic reprogramming open up intriguing possibilities for a broader impact on epigenetic regulation. The reduction in acetyl-CoA, a key substrate for histone acetyltransferases, could lead to a global decrease in histone acetylation, leading to transcriptional repression. Conversely, the surge in lactate production raises the possibility of increased histone lactylation, a recently discovered histone modification linked to the activation of genes involved in wound healing and M2-like polarization of macrophages (52, 53). This potential switch from histone acetylation to lactylation, possibly regulated by ZMYND8, could represent a novel mechanism by which metabolic reprogramming directly influences the epigenetic landscape and gene expression patterns in cancer cells.

The correlation between ZMYND8 and HIF-1α expression in patient samples, particularly in luminal breast cancers, highlights the potential clinical relevance of our findings. This association suggests that ZMYND8 could serve as a prognostic marker or therapeutic target, especially in hypoxic tumors. Moreover, the impact on immune cell invasion raises the possibility that targeting ZMYND8 could enhance the efficacy of immunotherapies in breast cancer.

In conclusion, our study identifies ZMYND8 as a key epigenetic regulator of the hypoxic response, bridging oxygen sensing with metabolic reprogramming. This represents a new paradigm in understanding how cancer cells adapt to and survive in low-oxygen environments. Our work elucidates a previously unrecognized mechanism by which epigenetic factors can influence the tumor immune microenvironment through metabolic reprogramming. The ZMYND8-mediated increase in lactate production and its subsequent impact on immune cell invasion provide a novel link between epigenetics, metabolism, and immune evasion in cancer. These findings not only advance our understanding of cancer metabolism but also open new avenues for therapeutic interventions targeting the epigenetic regulation of metabolic adaptation in tumors.

## Experimental procedures

### Cell culture

T47D and MCF7 (luminal breast cancer), MDA-MB-468 (triple negative breast cancer), HEK293T cells procured from the National Centre for Cell Science, Pune and the American Type Culture Collection. MCF7, MDA-MB-468, and HEK293T were cultured in complete Dulbecco's modified Eagle's medium (DMEM) (1 g/L glucose) supplemented with 10% fetal bovine serum, anti-anti, glutamax, and kept in a 5% CO2 incubator under humidified conditions. T47D was cultured in complete RPMI1640 (1 g/L glucose) supplemented with 10% fetal bovine serum, anti-anti, glutamax, and kept in a 5% CO2 incubator under humidified conditions.

### Transfection for knockdown and overexpression study

ZMYND8 shRNA (Santa Cruz Biotechnology, sc-76337-SH) and FLAG-tagged ZMYND8 full-length plasmids were transfected with the method described elsewhere (54, 55). Briefly, plasmid DNA was transfected using FuGENE HD reagent (Promega, #E2311) according to the manufacturer's protocol. Cells were seeded so that the next day 70 to 80% confluency could be reached after 24 h during transfection. Optimum amount of plasmid DNA diluted in serum-free medium has been incubated with FuGENE HD reagent and incubated for 20 min at room temperature (RT), and subsequently applied to the cells, and results were visualized after 24 h.

### Human patients' sample

Human breast cancer patient tissue specimens were collected with the consent of the patients from the multidisciplinary research unit at RG. Kar Medical College and Hospital, Kolkata, India, for this prospective study. Molecular subtyping has been done for the patients and characterized as ER+/PR+/HER2 (Low). Clinical details of the patients were recorded, including stage, grade, and tumor, node, and metastasis (TNM) (Table S4). The research work, along with the analysis, was performed at the Saha Institute of Nuclear Physics, Kolkata, India, as this study was approved by the Institutional Ethics Committee (ECR/322/inst/WB/2013/RR-20).

### Hypoxia treatment

For hypoxia treatment, cells were seeded in six-well or 60 mm plates 1 day before the treatment. On the day of treatment, 70 to 80% confluent dishes were placed on the rack of the hypoxia chamber (Stem-cell technologies), and the chamber was purged with mixed gas containing 5% CO2, 1% O2, and 94% inert N_2_ under 20 psi pressure for 5 min.

### RNA isolation and qRT-PCR

RNA from the cell was isolated by using TRIzol (HiMedia), followed by chloroform and isopropanol wash. Two milligrams of the total RNA was reverse-transcribed using a Thermo Fisher Scientific cDNA Synthesis kit as per Manufacturer's protocol. The complementary DNAs were then subjected to real-time PCR using SYBR Green Master mix in Applied Biosystems real-time PCR system by using an appropriate primer (Table S2). Relative gene expression was calculated using ΔΔCt method with 2−ΔΔCt as fold change.

### mRNA stability assay

A published protocol was followed (56). Briefly, 3 × 10ˆ^5^ cells were seeded per well in 3 ml of media in each well of a six-well plate. After 24 h, cells were transfected with ZMYND8 shRNA, and cells were kept in 5% CO_2_ conditions. Hypoxic condition was maintained by keeping the cells in a hypoxia chamber for 24 h. The next day, t = 0, cells were harvested using trypsinization, and RNA was extracted using the TRIzol method. The rest of the cells were treated with actinomycin D so that the final concentration reached 10 μg/ml. Samples were harvested at 1,2,3,4, and 5 h, respectively. qRT-PCR was performed from the extracted RNA, and the average Ct values of t-0 were subtracted from each time point to obtain the ΔCt value.

ΔCt = (Average Ct of each time point-Average Ct of t = 0). Relative abundance of mRNA at each time point was calculated using the formula mRNA abundance = 2^(-ΔCT).

Relative abundance of mRNA at each time point relative to t = 0 was plotted using GraphPad Prism. mRNA half-life was calculated by calculating the mRNA decay rate by nonlinear regression curve fitting (one-phase decay) using GraphPad Prism. The following parameters were used: least squares (ordinary fit), confidence level–95%, asymmetrical (likelihood) CI, goodness-of-fit was quantified with R square, convergence criteria–medium.

### Western blotting

For whole cell lysates, cells were homogenized in standard radioimmunoprecipitation assay (RIPA) buffer, supplemented with 1X PIC and 1X PhoSTOP. Protein concentration was estimated using the Bradford assay. Subsequently, 80 to 100 μg of protein was mixed with sample loading buffer and heated at 95 °C for 2 to 3 min before 11% SDS-PAGE gel running. The proteins were transferred to a nitrocellulose membrane, blocked with 5% nonfat milk in 1X TBS-Tween 20 solution before respective primary antibody binding overnight (Table S1). Next, the membranes were probed with respective horseradish peroxidase-tagged secondary antibodies and visualized by chemiluminescence. Western blot band intensities were quantified using image J software.

### 3D spheroid culture

For 3D sphere culture, 2.5 to 4 × 10^3^ cells per well were seeded in a 6 well Ultra-Low Attachment plate (Corning) in serum-free DMEM/F12 medium supplemented with insulin (5 ug/ml), recombinant epidermal growth factor (20 ng/ml), B27 supplement, and 0.4% bovine serum albumin (BSA) basic fibroblast growth factor (20 ng/ml). The spheres were maintained undisturbed and without media changing for 7 to 10 days. The serum-free media was gently added every 2 days to the wells dropwise.

### Immunofluorescence staining of MCTS

Multi cellular tumor spheroids were fixed with 4% para formaldehyde for 10 min after discarding the culture media and washing 2 times with PBS. The MCTS were permeabilized with 0.1% Triton X-100 for 20 min, and MCTS were subsequently blocked by 3% BSA for 1 h. Primary antibodies according to mentioned dilution (Table S1) were applied overnight. The next day, primary antibody was discarded and MCTS were washed very gently twice with PBS and secondary antibody was applied for 1 h at RT. Subsequently, MCTS were counterstained with 4′,6-diamidino-2-phenylindole. MCTS were then carefully transferred on to slides and mounted with DPX medium and air-dried overnight before proceeding for microscopic imaging.

### Primary human tissue sample collection

The breast tumor tissue samples along with informed patient consent and patient details were obtained from RG Kar Medical College and hospital with approval to institutional bio-ethics committee (RG Kar MCH).

### Immunohistochemistry

Formalin-fixed and paraffin embedded blocks (obtained from RG Kar Medical College and hospital, Kolkata) sectioned using microtome. Four micrometer sections are mounted on Poly L-lysine coated slides. Slides are heated at 65 °C for 40 min and placed in xylene for deparaffinization. Then slides are rehydrated by placing them sequentially in 100%, 90%, 80%, and 70% ethanol for 3 to 5 min in each step. Subsequently, slides are placed in fresh distilled water for 10 min. Slides were placed in an antigen retrieval buffer and heat-mediated antigen retrieval was done in microwave heating for 10 min in high power and subsequently in low power for 10 min. Slides were cooled to RT and then immune-staining was done according to manufacturer's protocol. Briefly, at first hydrogen peroxide block was applied to block endogenous peroxidase activity followed by a protein block was used for 10 min each. Subsequently, the slides were incubated with respective primary antibodies (Table S1) for 60 min followed by secondary antibody binding for 15 min at RT. 3,3'-diaminobenzidine chromogen was applied, and incubated for 1 min in the dark, then washed immediately before counterstaining with hematoxylin and mounting using DPX mounting solution.

For tissue microarray (# BR1507) staining, the microarray slide was baked at 60 °C for 1 h and similar procedure was followed.

### Chromatin immunoprecipitation

ChIP assay followed by qRT-PCR was performed as previously described protocol (57). Briefly, the cell was incubated with 1% formaldehyde for 10 min at RT, and 125 mM glycine was used to terminate the reaction for 10 min at RT. Firstly, the cell lysis buffer (Farnham's lysis buffer) (5 mM Pipes pH 8.0, 85 mM KCl, 0.5% NP-40) with 1X PIC was used to resuspend and lyse the cells. The intact nuclei were isolated by centrifuging at 3000 rpm for 5 min at 4 °C, followed by resuspension in nuclei ChIP lysis buffer (50 mM Tris–HCl pH 8.0, 10 mM EDTA, and 1% SDS). Following this, the chromatin was sheared using a sonicator, and immunoprecipitation was performed using Dyna beads blocked in 5% BSA (Thermo Fisher Scientific) for 3 h. The immunoprecipitant bead was pelleted down by centrifuging at 5000 rpm at for 5 min at 4 °C. Immunoprecipitant beads were washed with RIPA buffer, high salt buffer, LiCl buffer, and TE buffer, respectively. The complex was then decrosslinked by heating at 65 °C overnight. The beads were then subjected to RNase A digestion followed by Proteinase K treatment to wash off all the RNA and protein from the beads leaving only the DNA fragments. Next, the phenol–chloroform extraction method was applied to purify and enrich the ChIP DNA. ChIP DNA was subjected to ethanol precipitation using isopropanol and sodium acetate for 48 h at −80 °C. Precipitated ChIP DNA was washed in 75% ethanol and air-dried. Subsequently, DNA was dissolved in RNase-free and DNase-free water and analyzed using specific primers *via* qPCR (Table S3).

### Coimmunoprecipitation

Cells harvested after the experiment and lysates were prepared using RIPA buffer and sonicated briefly for proper lysis and homogenization. The supernatant is collected *via* centrifuging at 13,000 RPM for 10 min at 4 °C. Lysates are precleared to avoid nonspecific interaction. Immunoprecipitation was set up using the respective antibodies. Immunoprecipitant beads are collected by centrifuging at 5000 rpm for 5 min at 4 °C. The beads are washed three times with RIPA buffer, with intermediate vortexing and rotation for 10 min. Finally, protein complexes are eluted by heating the beads with gel-loading buffer at 95 °C for 10 min. The SDS page was performed using the samples, followed by western blotting.

### Extracellular acidification rate measurement

ECAR measurement was done using the extracellular flux analyzer XFp by Seahorse Biosciences (Agilent Technologies) following our previously published protocol (57). In brief, cells were cultured in an XFp cell culture plate in complete DMEM media for 24 h. One hour before the measurement, the cells were washed with ECAR media (XF base medium minimal DMEM, 102353-100, supplemented with 1 mM sodium pyruvate, 2 mM glutamax, and adjusted to pH 7.4). Then they kept in ECAR media in a 37 °C CO2-free incubator. Meanwhile, the overnight hydrated XFp cartridge was loaded with 10 mM glucose, 1.5 μM oligomycin A, and 15 μM 2-DG in Port A, Port B, and Port C, respectively, and then the cartridge was loaded for calibration. After calibration, the cartridge plate was replaced with a cell plate, and the ECAR measurement proceeded. The data were analyzed and plotted using Wave Desktop 2.6.3 by Agilent Technology and GraphPad Prism 8.4.2.

### OCR measurement

OCR measurement was done using an extracellular flux analyzer XFp by Seahorse Biosciences (Agilent Technologies), following our previously published protocol (58). In brief, cells were cultured in an XFp cell culture plate in complete RPMI-1640 media for 24 h in a 37 °C 5% CO_2_ incubator. One hour before the measurement, the cells were washed with OCR media (XF Base Medium Minimal DMEM, 102353-100, supplemented with 1 mM sodium pyruvate, 2 mM glutamax, 5.5 mM glucose, adjusted pH 7.4) and kept in OCR media in a 37 °C CO2-free incubator. Meanwhile, the overnight hydrated XFp cartridge was loaded with 1.5 μM oligomycin A, 1 μM carbonyl cyanide 4-(trifluromethoxy)phenylhydrazone, and 1 μM rotenone/Antimycin A in Ports A, B, and C, respectively, and then calibrated. After calibration, the cartridge plate was replaced with a cell plate, and the OCR measurement proceeded. The data were analyzed and plotted using Wave Desktop 2.6.3 by Agilent Technology and GraphPad Prism 8.4.2.

### Measurement of UK-5099 mediated acute response

For measuring UK5099 dependent acute response OCR measurement was done using an extracellular flux analyzer XFp by Seahorse Biosciences (Agilent Technologies), following manufacturer protocol. In brief, cells were cultured in an XFp cell culture plate in complete RPMI-1640 media for 24 h in a 37 °C 5% CO_2_ incubator. One hour before the measurement, the cells were washed with OCR media (XF Base Medium Minimal DMEM, 102353–100, supplemented with 1 mM sodium pyruvate, 2 mM glutamax, 5.5 mM glucose, adjusted pH 7.4) and kept in OCR media in a 37 °C CO2-free incubator. Meanwhile, the overnight hydrated XFp cartridge was loaded with 2 μM UK-5099, 1.5 μM oligomycin A, 1 μM carbonyl cyanide 4-(trifluromethoxy)phenylhydrazone, and 1 μM rotenone/Antimycin A in Ports A, B, and C and D respectively, and then calibrated. After calibration, the cartridge plate was replaced with a cell plate, and the OCR measurement proceeded. The data were analyzed and plotted using Wave Desktop 2.6.3 by Agilent Technology and GraphPad Prism 8.4.2.

### PBMC invasion assay

PBMC was isolated from blood (from consented patients registered to KPC Medical College and Hospital, Kolkata; clearance from the hospital was sought on 12.06.2024) by using HiSep LSM1077 (HiMedia). Control, hypoxia-treated, and hypoxia-treated in ZMYND8 knockdown background T47D cell media were collected and centrifuged at 3000 rpm for 10 min to remove the cellular debris. This medium was added to the lower chamber of the trans healthy plate (Thermo Fisher Scientific). The trans-well membrane was coated with Geltrex extracellular matrix for 60 min and washed with PBS. The PBMC was seeded on the upper chamber in incomplete DMEM media and incubated for 24 h in a 5% CO_2_ incubator at 37 °C. To measure the invading PBMC amount, the media from the upper chamber were discarded gently and washed with PBS three times and then fixed with 4% paraformaldehyde for 15 min. The cell was visualized by staining with 2% crystal violet for 20 min at RT. The image was captured in a brightfield light microscope under a 20X objective lens.

### Human T cell culture

PBMCs were obtained from buffy coats of healthy human donors through Ficoll-Hypaque density gradient centrifugation. Naive CD8 T cells were then isolated using magnetic bead purification and stimulated for 72 h with plate-bound anti-CD3 (5 μg/ml) and anti-CD28 (5 μg/ml) in the presence of T47D cell line-derived media along with recombinant human IL2 (100U/ml).

For the preparation of conditioned media, the T47D cell line was cultured in complete RPMI media under normoxic or hypoxic conditions. After 24 h of culture, the media were collected and centrifuged at 3000 rpm for 10 min to remove cellular debris and stored for T cell culture.

### Flow cytometry

Three-days activated human T cells were restimulated for 6 h using PMA, ionomycin, and Golgi-Plug, either in complete RPMI medium or in the same conditioned media used during the initial activation. Cell surface marker staining was carried out before fixation and permeabilization using the BD Cytofix/Cytoperm Kit (BD Biosciences). All antibodies were diluted at 1:200 in fluorescence-activated cell sorting buffer (composed of 0.5% BSA and 0.1% sodium azide in PBS) and incubated with the cells for 30 min at 4 °C. Data acquisition was performed on a BD LSRFortessa flow cytometer, and analysis was conducted using FlowJo software (BD Biosciences).

### Animal study

Six- to eight-week-old female BALB/c mice (animal ethics clearance was sought from the Department of Biochemistry, University of Calcutta; Registration number: 797/GO/Re/S/03/CPCSEA; dated: 05.09.2019) were procured and quarantined for a week in individual ventilated cages under alternate dark and light cycles and maintained on food and water in the central animal house facility of University of Calcutta (CU). Animals were injected with 3 × 10^6^ either WT or ZMYND8 overexpressed (ZMYND8 O/E) 4T1 cells suspended in 0.1-ml PBS were injected (subcutaneously) into right side of the flank. After 7 to 10 days, primary tumors were visible. Animals were sacrificed, and tumors were harvested on the 28th day from the injection.

### Patient RNA-sequencing data analysis

RNA-sequencing data of patients' breast cancer samples, Breast Cancer (METABRIC, Nature 2012 & Nat Commun 2016) (37, 38) was analyzed by cBioPortal web server. From 2509 primary breast tumors, the sample was shortened for the group ER+/PR+/HER2-with no therapeutic intervention. The ZMYND8 correlated genes and their expression values were downloaded, and a KEGG pathway analysis was performed using ShinyGO 0.80. The network analysis was performed using Cytoscape 3.8.2 and its built-in GeneMANIA. The correlation plot was done using GraphPad prism 8.4.2.

### Cellular Acetyl-CoA measurement

The amount of cellular acetyl-CoA was measured by using an Acetyl-CoA measurement assay kit (antibodies.com, A319654) following the manufacturer's protocol. In brief, an equal number of cells were lysed using acetyl-CoA lysis buffer, and 15 μl of the lysate was used to set the reaction. The absorbance was measured at 340 nm in a 96-well plate reader.

### Cellular lactate measurement

The amount of cellular lactate was measured using a lactate measurement assay kit (Abcam, ab65331) according to the manufacturer's protocol. In brief, an equal number of cells was lysed using lactate lysis buffer. The sample was deprotonated using 4 M perchloric acid, and the pH was adjusted to 6.5 to 8.0 with 2 M KOH. The dilution factor was notated for calculation. Thirty microliters of the lysate was used to set the reaction. The absorbance was measured at 450 nm in a 96-well plate reader.

### Extracellular lactate measurement

The amount of extracellular lactate was measured using a lactate measurement assay kit (Abcam, ab65331) according to the manufacturer's protocol. In brief, the cell media was collected and deprotonated using 4 M perchloric acid, and the pH was adjusted to 6.5 to 8.0 using 2 M KOH. Then the media was concentrated for 5 h in a 4 °C concentrator. The dilution factor and the concentrating factor were notated for calculation. Twenty microliters of the lysate was used to set the reaction. The absorbance was measured at 450 nm in a 96-well plate reader.

### Statistical data analysis

All the statistical data were analyzed by using GraphPad Prism 8.4.2. For a data set with only two variables, an unpaired *t* test was performed, and for more than two variables, a one-way ANOVA was performed. For the correlation analysis, Pearson's simple regression analysis was done. The error bar represents the Standard Error of Mean (SEM).

## Data availability

All the data are provided in the main manuscript or the supporting information file.

## Supporting information

This article contains supporting information.

## Conflict of interest

The authors declare that they have no conflicts of interest with the contents of this article.
