# Supplementary material for: The hypoxia-induced chromatin reader ZMYND8 drives HIF-dependent metabolic rewiring in breast cancer
Source: J Biol Chem. 2025 Sep 3;301(10):110680. doi: 10.1016/j.jbc.2025.110680 (PMC12516554; doi:10.1016/j.jbc.2025.110680)
Supplement: Supporting information [file mmc1.docx]

**Supporting information**

**The Hypoxia-Induced Chromatin Reader ZMYND8 Drives**

**HIF-Dependent Metabolic Rewiring in Breast Cancer**

Sandhik Nandi^1,2,#^, Atanu Mondal^1,2,#^, Ishita Sarkar^3^, Md Wasim Akram Ddoza Hazari^1,2^, Indrakshi Banerjee^1,2^, Shantanu Ghosh^4^, Himansu Roy^5^, Abhra Banerjee^6^, Anjali Bandyopadhyay^7^, Shritama Aich^6^, Sanghamitra Sengupta^4^, Shilpak Chatterjee^3^, Chandrima Das^1,2,^*

^1^Biophysics and Structural Genomics Division, Saha Institute of Nuclear Physics, 1/AF Bidhannagar, Kolkata 700064, India.

^2^Homi Bhabha National Institute, Anushakti Nagar, Mumbai 400094, India.

^3^Division of Cancer Biology and Inflammatory Disorder, IICB-Translational Research Unit of Excellence, CSIR–Indian Institute of Chemical Biology, Kolkata 700032, India.

Academy of Scientific and Innovative Research (AcSIR), Ghaziabad 201002, India.

^4^Department of Biochemistry, University of Calcutta, Kolkata, West Bengal, India

^5^Department of Surgery, KPC Medical College and Hospital, Kolkata, West Bengal, India.

^6^Multi-Disciplinary Research Unit, R.G.Kar Medical College and Hospital, Kolkata, West Bengal, India.

^7^Department of Pathology, R.G.Kar Medical College and Hospital, Kolkata, West Bengal, India

# Contributed equally.

*Correspondence:

Chandrima Das, Ph.D.

Biophysics and Structural Genomics Division,

Saha Institute of Nuclear Physics,

1/AF Bidhannagar, Kolkata 700064, India

E-mail: [chandrima.das@saha.ac.in](mailto:chandrima.das@saha.ac.in)

Phone: +91-9830185993


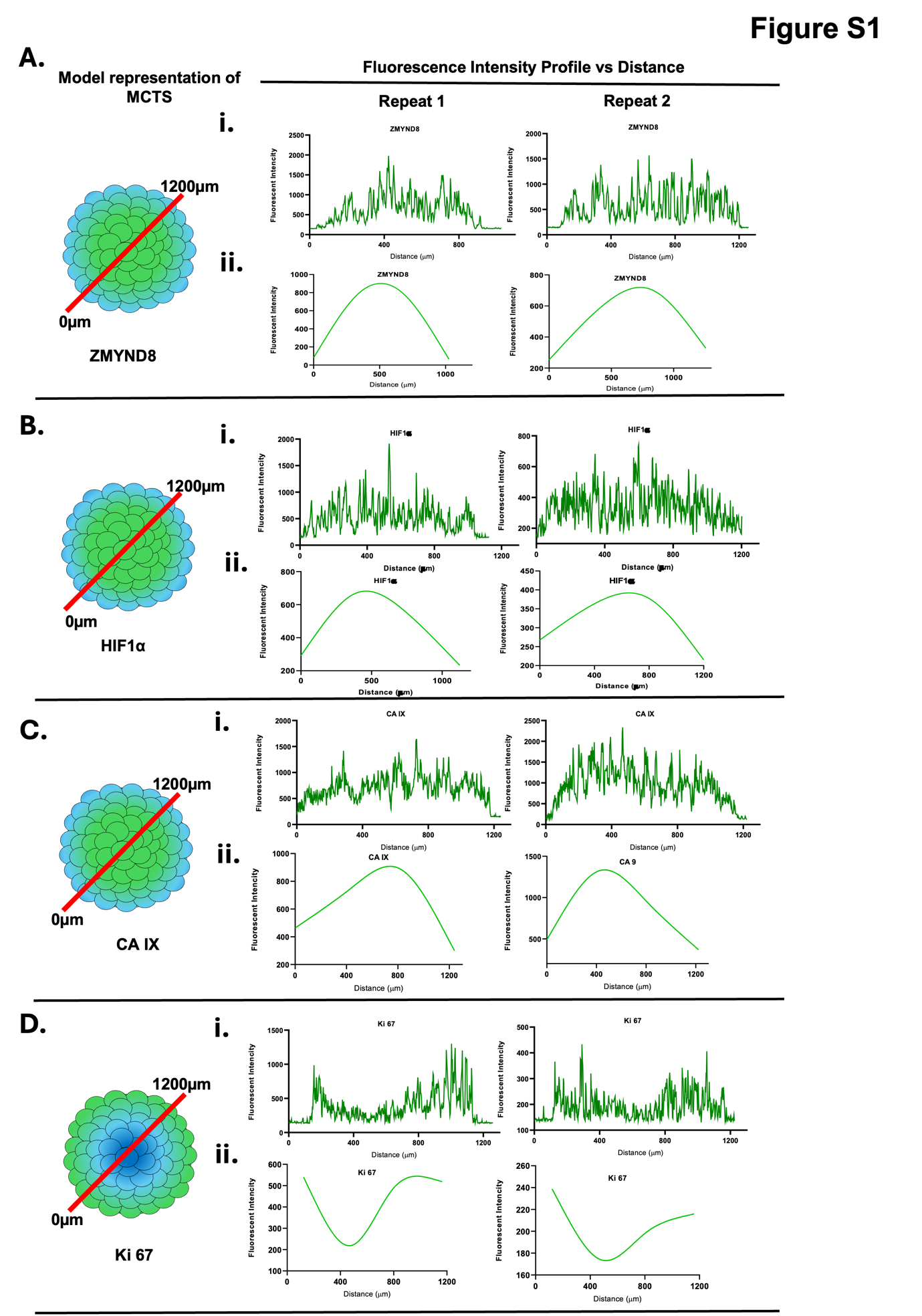


**Fig S1: ZMYND8 and HIF1-α expressed at the centre of 3D MCTS**. (A-D) Fluorescence intensity profile (i) and “Fit Spline” representation (ii) of ZMYND8, Hif1⍺, CAIX, and Ki67 in MCTS as a function of the indicated distance.

**
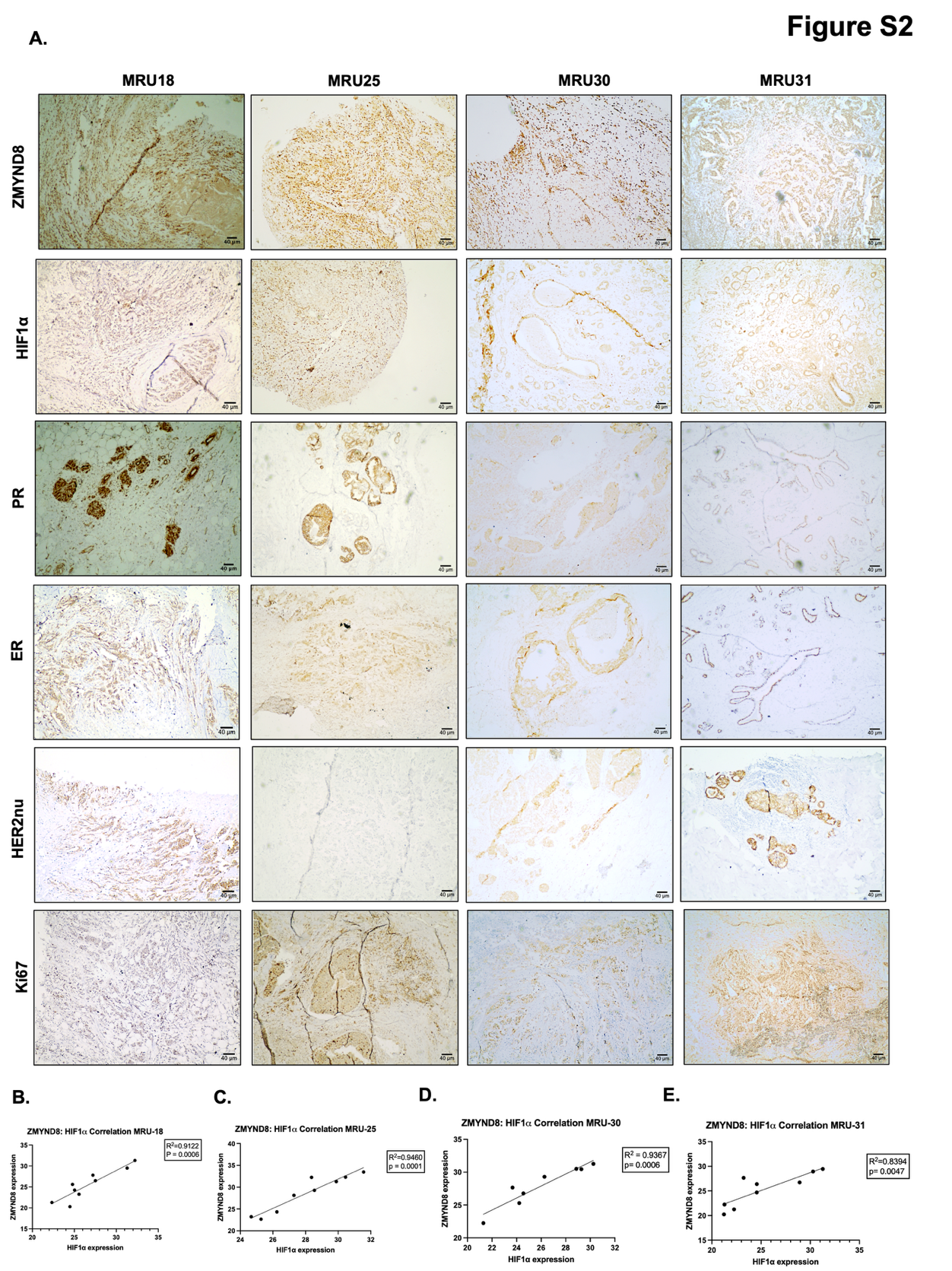
**

**Fig S2: Immunohistochemistry of ZMYND8 HIF1⍺ in breast cancer patients** (A) Immunohistochemical staining of ZMYND8, HIF1⍺, ER,PR,HER2 and Ki67 from breast tumors. (B-E) Correaltion graph showing quantification of multiple regions of immunohistochemical staining for every indiviual tumor. Correlation plot of ZMYND8 and HIF1α absolute expression values. Pearson correlation analysis was performed to calculate the p-value significance and simple linear regression analysis was done to calculate the R value.


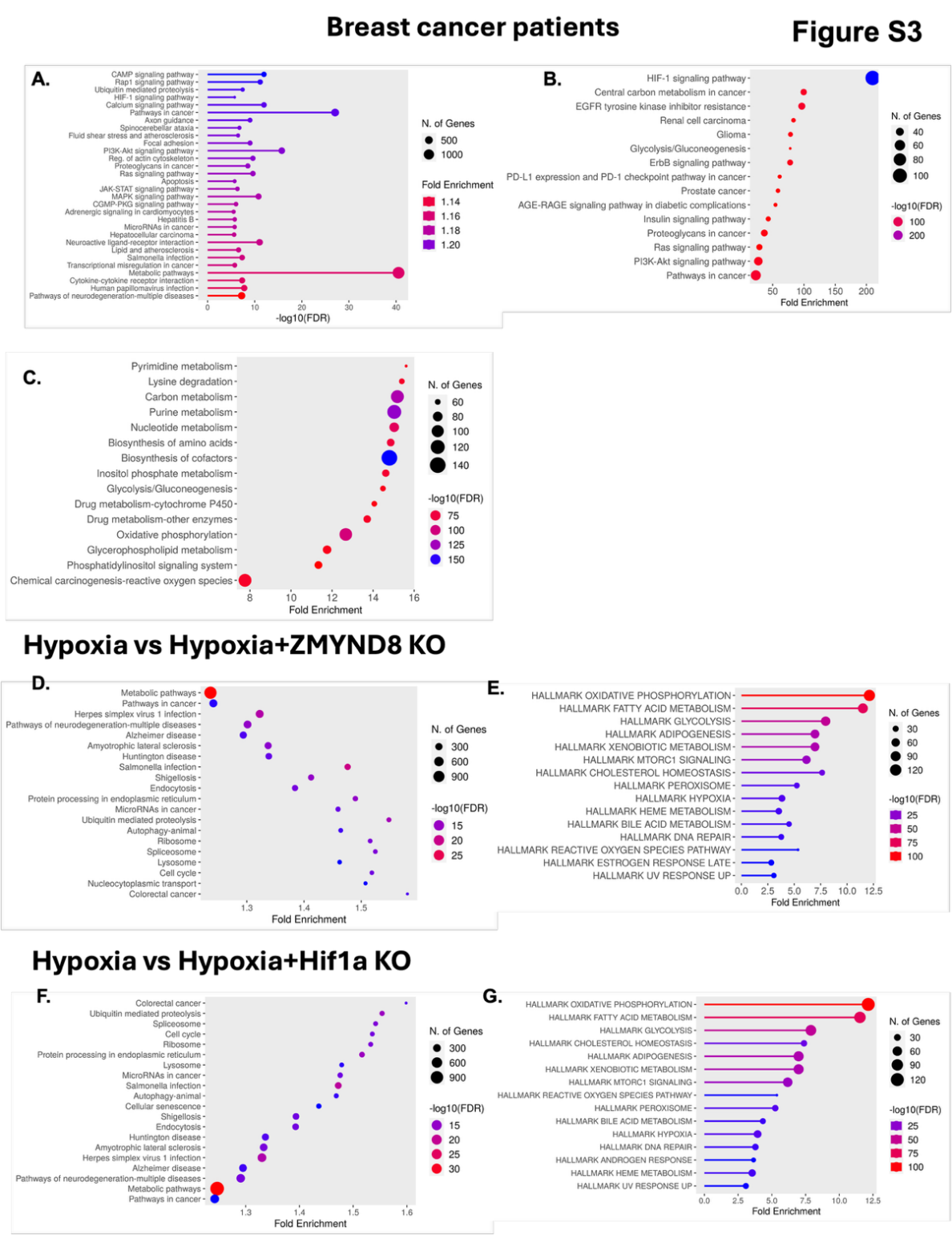


**Fig S3: ZMYND8 and HIF1⍺ regulates breast cancer transcriptome** (A) KEGG pathway analysis of ZMYND8 and HIF1⍺ correlated genes using ShinyGo webserver. (B) KEGG pathway analysis of subset of genes under ‘metabolic processes’ using ShinyGo webserver. (C) Biological process analysis of genes under ‘metabolic process’ using ShinyGo webserver. (D-E) Biological process analysis and Hallmark DB analysis of singificant genes (FC>1.5) from ZMYND8 knockout DEGs in MDA-MB-231 cells respectively. (F-G) Biological process analysis and Hallmark DB analysis of singificant genes (FC>1.5) from HIF1⍺ knockout DEGs in MDA-MB-231 cells respectively.

**
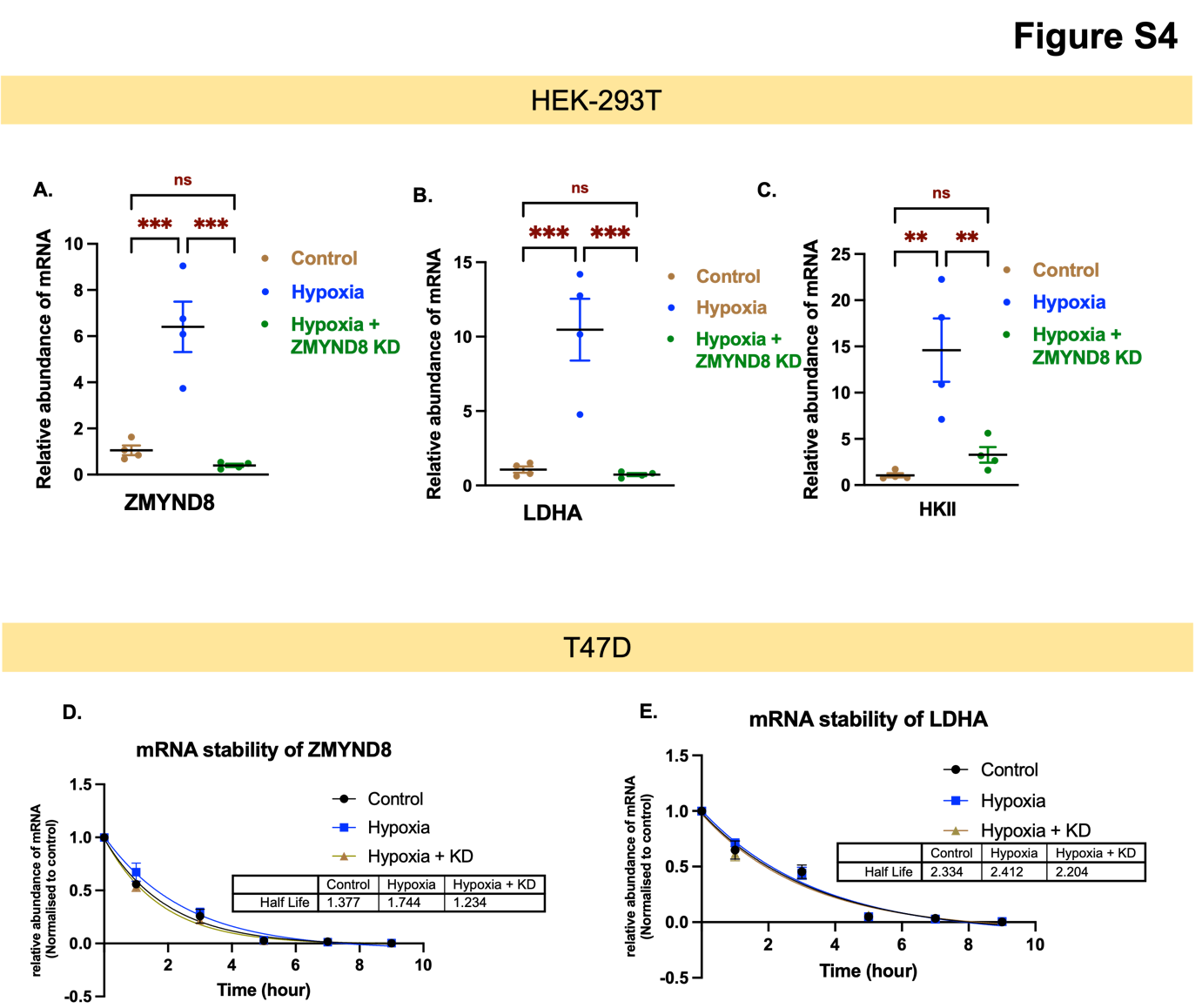
**

**Fig S4: ZMYND8 regulates LDHA and HKII transcription** (A-C) qRT-PCR assay from HEK293T cells showing ZMYND8, LDHA and HK2 expression under control, hypoxia and ZMYND8 knockdown in hypoxic conditions. One-way ANOVA was performed to analyse the p-value significance (*p<0.05; **p<0.01; ***p<0.001; ns, non-significant (p>0.05)) for the statistical analyses, and the error bar represents the standard error of the mean (SEM). (D-E) mRNA stability assay showing half-life of ZMYND8 and LDHA mRNA.


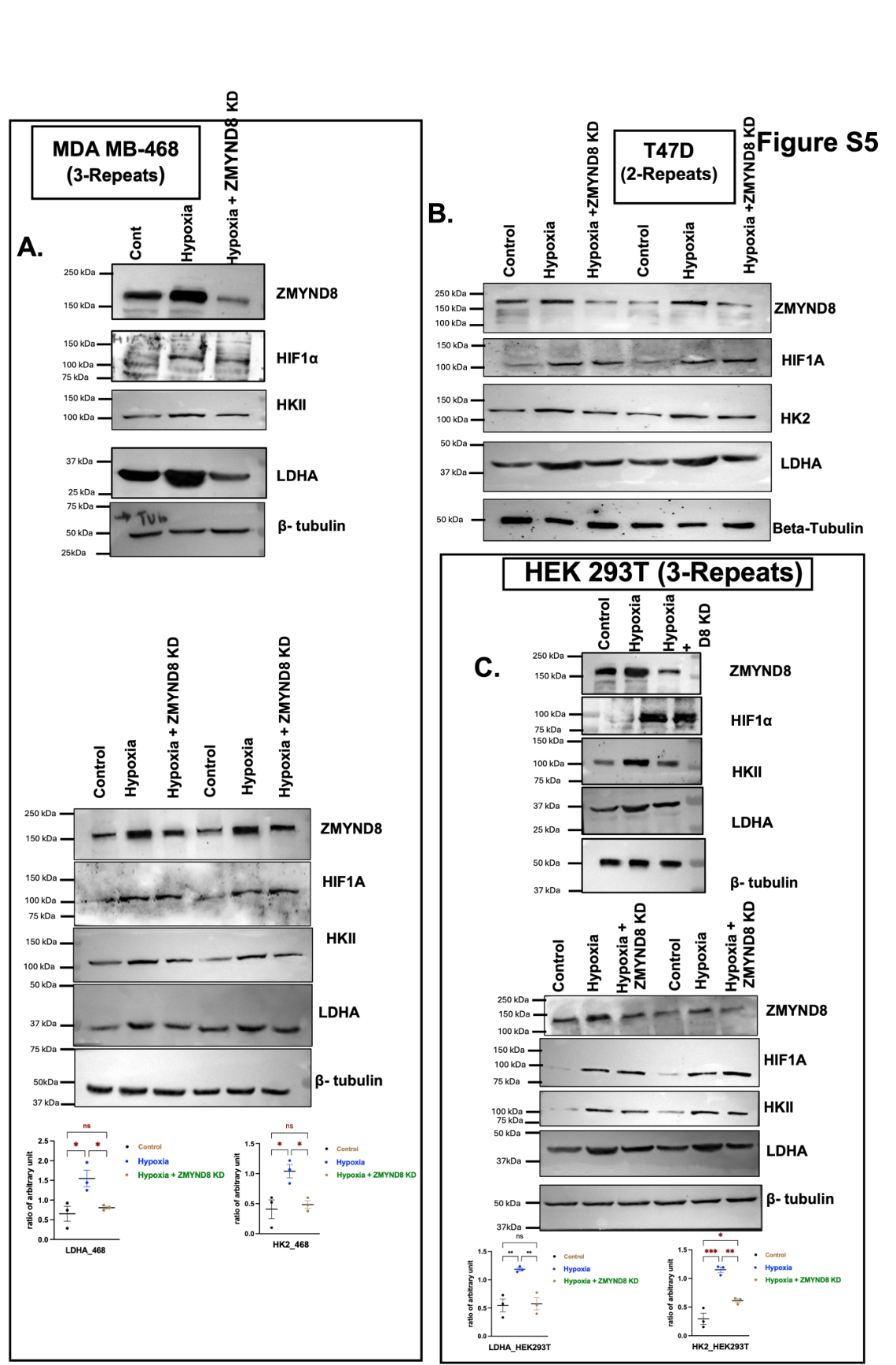
**Fig S5: ZMYND8 regulates LDHA and HK2 protein level expression in different cell lines** (A) Western blot images showing expression of LDHA, ZMYND8 and HIF1⍺ in MDA-MB-468 cells. Graphs showing densitometric quantification of western blot band intensities. One-way ANOVA was performed to analyse the p-value significance (*p<0.05; **p<0.01; ***p<0.001; ns, non-significant (p>0.05) for the statistical analyses, the error bar represents the standard error of the mean (SEM). (B) Western blot images showing expression of LDHA, ZMYND8 and HIF1⍺ in T47D cells. (C) Western blot images showing expression of LDHA, ZMYND8 and HIF1⍺ in HEK-293T cells. Graphs showing densitometric quantification of western blot band intensities. One-way ANOVA was performed to analyse the p-value significance (*p<0.05; **p<0.01; ***p<0.001; ns, non-significant (p>0.05) for the statistical analyses, the error bar represents the standard error of the mean (SEM).

**
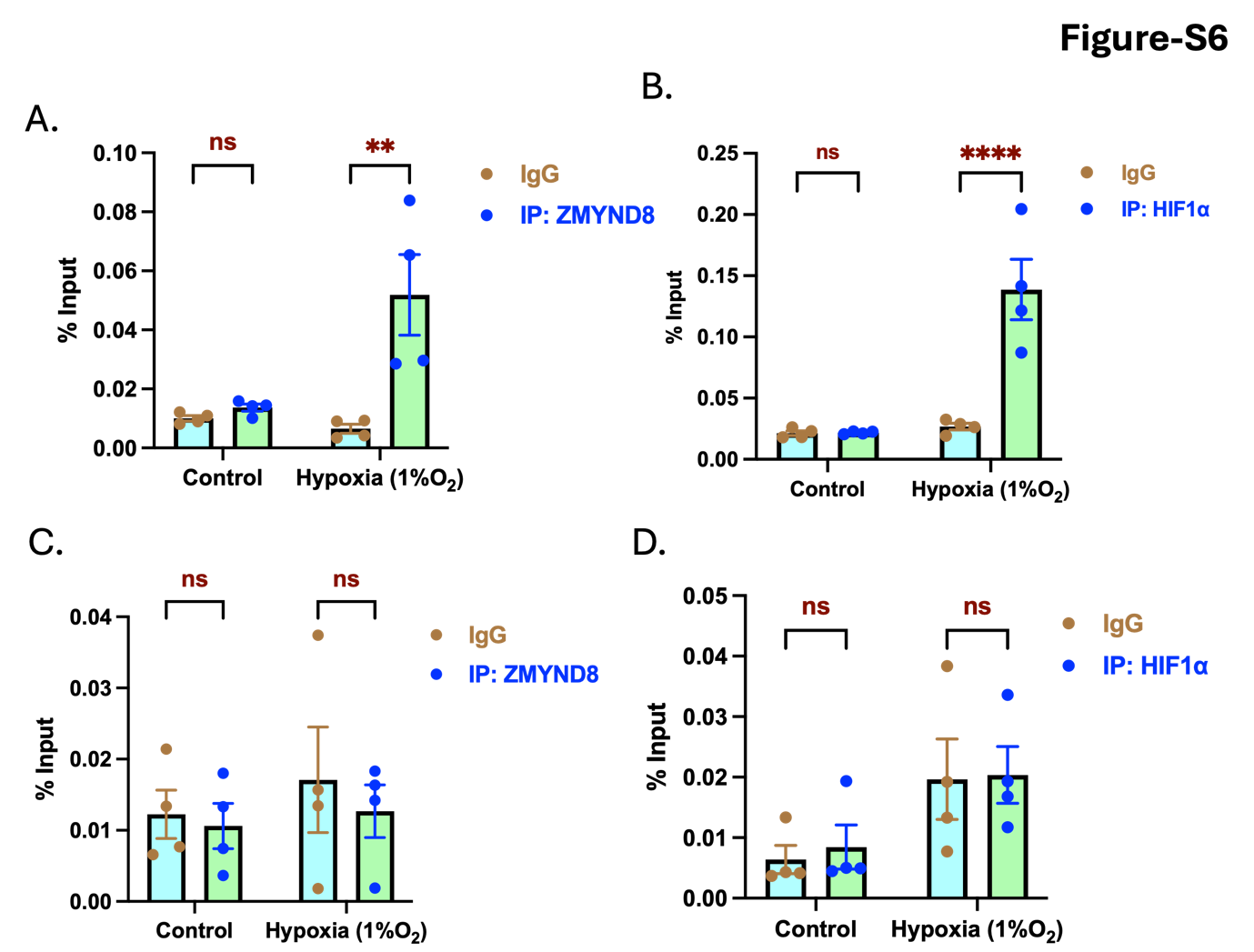
**

**Fig S6: ZMYND8-HIF1𝛼 co-recruitment regulates LDHA gene transcription** (A-B) ChIP qPCR assays showing recruitment of ZMYND8 and HIF1⍺ in control and Hypoxic conditions in the LDHA-HRE region 2. (C-D) ChIP qPCR assays showing recruitment of HIF1⍺ in the intergenic region (negative control) of the LDHA promoter in control and Hypoxic conditions. Two-way ANOVA was performed to analyse the p-value significance (*p<0.05; **p<0.01; ***p<0.001; ns, non-significant (p>0.05)) for the statistical analyses, and the error bar represents the standard error of the mean (SEM).


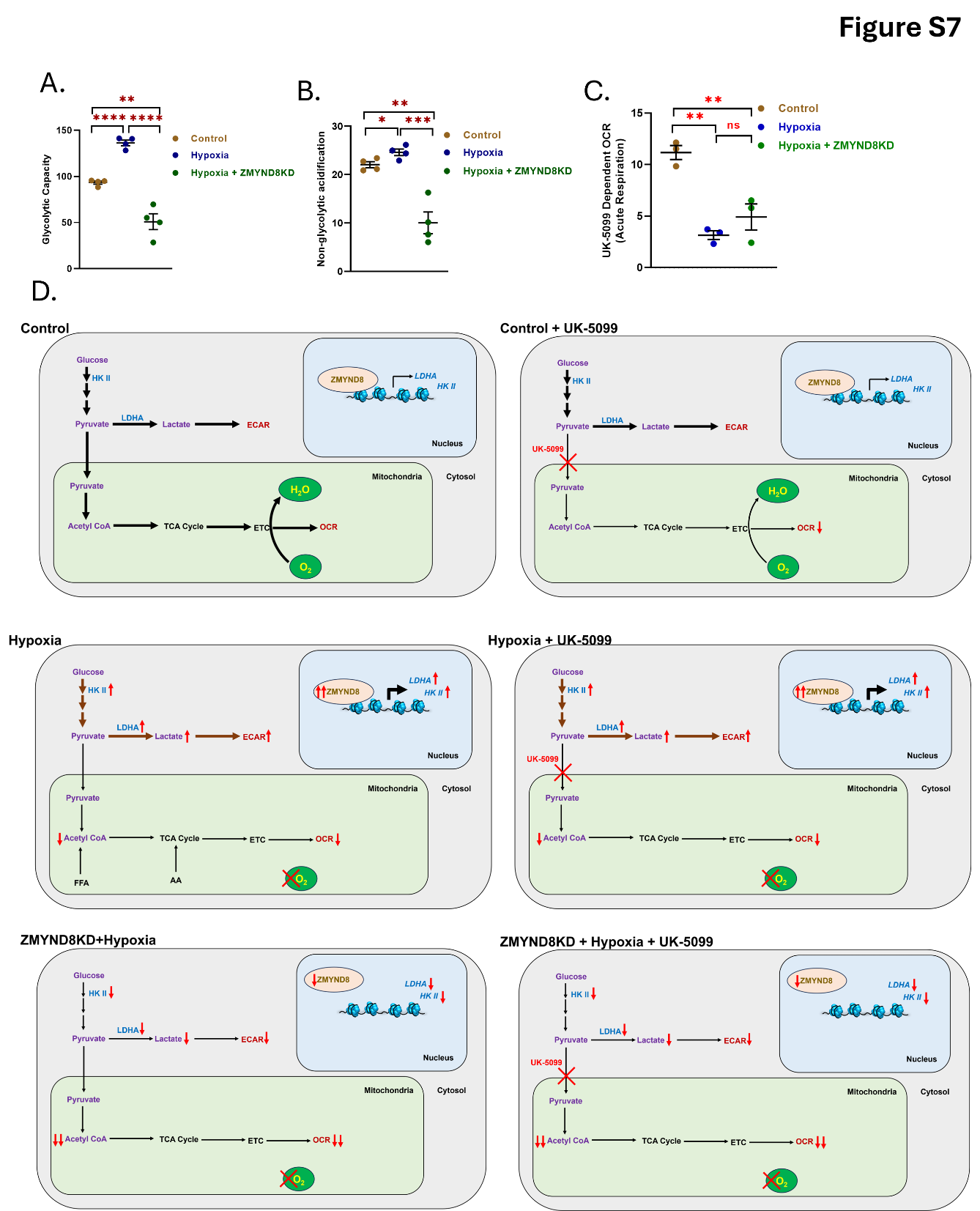


**Fig S7: ZMYND8 shifts metabolic flux towards lactate production upon hypoxia.** (A-C) Quantification of glycolytic capacity, non-glycolytic acidification and UK-5099-dependent OCR using Seahorse flux analyser in Control, Hypoxia and ZMYND8 knockdown in hypoxic conditions. One-way ANOVA was performed to analyse the p-value significance (*p<0.05; **p<0.01; ***p<0.001; ns, non-significant (p>0.05)) for the statistical analyses, and the error bar represents the standard error of the mean (SEM). (D) Graphical representation of the cellular phenomenon happening in control, Hypoxia and ZMYND8 knockdown conditions in the presence and absence of UK-5099.

**
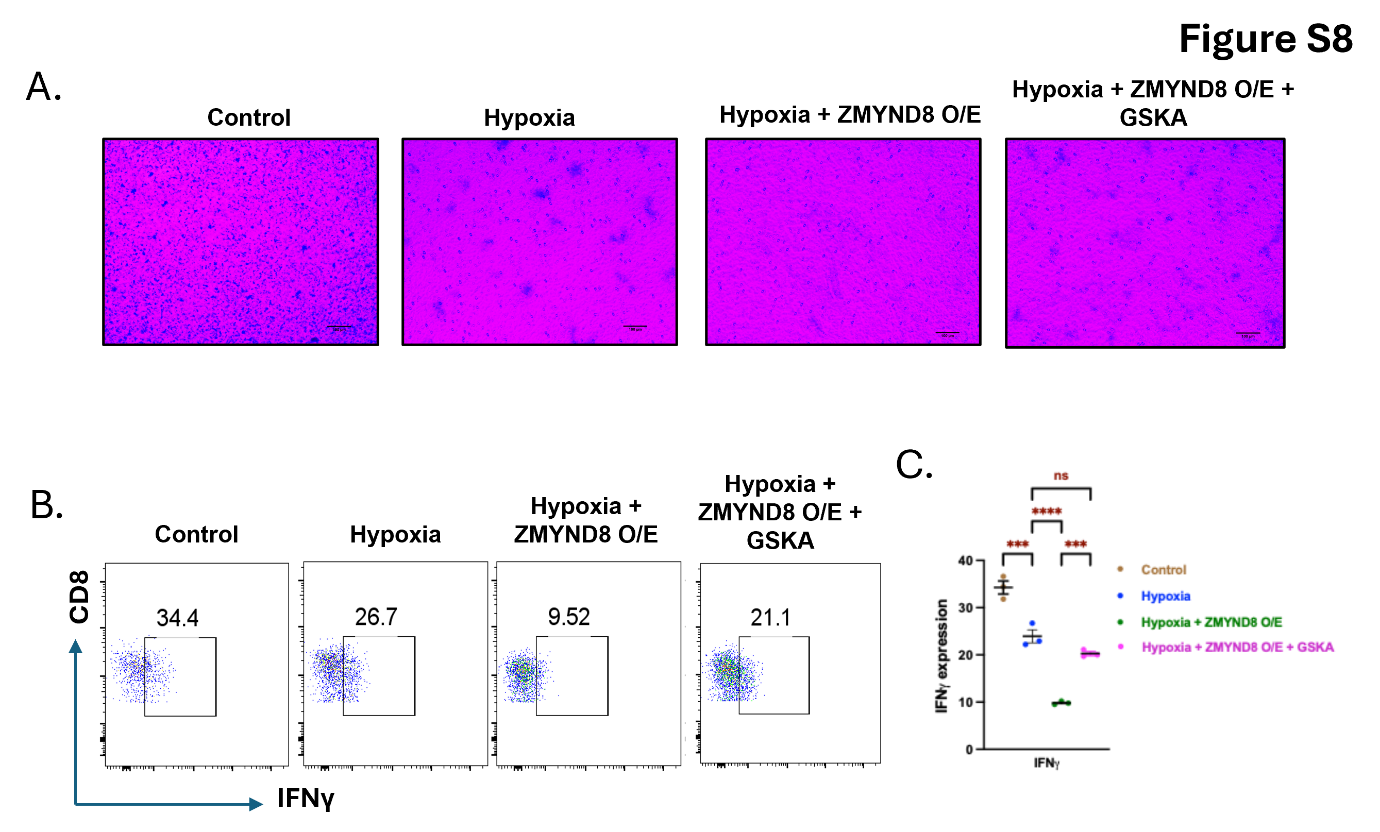
**

**Fig S8: ZMYND8 regulates PBMC invasion upon hypoxia.** (A) Bright-filed microscopy image of PBMC invasion through the matrix in the presence of conditioned medium from cells cultured in Control, Hypoxia and ZMYND8 overexpressed in hypoxic condition in presence and absence of GSKA. (B-C) Flow cytometry image and quantification showing percentage of IFNγ positive cells in control, hypoxia and ZMYND8 overexpression in hypoxic condition in presence and absence of GSKA. One-way ANOVA was performed to analyse the p-value significance (*p<0.05; **p<0.01; ***p<0.001; ns, non-significant (p>0.05)) for the statistical analyses, and the error bar represents the standard error of the mean (SEM).

**Key resource table**

**Table S1: Antibodies**

| **Antibody** | **Catalog Number** | **Dilution** |
| --- | --- | --- |
| LDHA | ab47010 | 1:1000 |
| CAIX | ab15086 | 1:1000 (Western blot)  1:100 (IF) |
| HIF1A | ab1 | 1:500 |
| Ki67 | ab 15580 | 1:1000 (Western Blot)  1:200 (IF) |
| ZMYND8 | HPA020949 | 1:1000 (Western Blot)  1:200 (IF) |
| ER | PR042 | Pre-diluted |
| PR | PR-068 | Pre-diluted |
| HER2/ErbB2-EP3 | PR047 | Pre-diluted |
| HK II | ab104836 | 1:1000 |
| Tubulin | ab6046 | 1:5000 |
| H3 | ab1791 | 1:10000 |
| HIF1A (for IF) | (ab51608) | 1:100 |
|  |  |  |

**Table S2: Primers**

| Primer | Forward (5’-3’) | Reverse (5’-3’) |
| --- | --- | --- |
| CAIX | AGTCATTGGCGCTATGGAGG | TCTGAGCCTTCCTCAGCGAT |
| 18S | GATTCCGTGGGTGGTGGTGC | AAGAAGTTGGGGGACGCCGA |
| ZMYND8 | CAGAAAATGAAACAGCCAGGG | ACTTTGCATCAGCCAGGAAG |
| LDHA | AGCCCGATTCCGTTACCT | CACCAGCAACATTCATTCCA |
| HKII | CTCAGAGCGGCTCAAGACAA | GCACACCTCCTTGACGATGA |
| HKII-mouse | GTCACACGAGGGATTTAG | CAGAGACACTCCCAATATAC |
| LDHA-mouse | CATTGCGTCCGTTGCAGATG | GGAGGAACAAGCTCCCGTG |
| ZMYND8-mouse | GGATATCTCTACTCGCTCC | GGTGAGTGGCTGCTTCATA |
| ACDH | GGGTGACAGTTCGTTAAG | GGGTGACAGTTCGTTAAG |
| VEGF | GACTAGAAGAGCTTGGATG | GACAAATACCAGGGTGAG |

**Table S3: ChIP Primers**

| LDHA-TSS-ChIP | CAAGCCTTCCTGAGAGTAACC | GGCCTTAAGTGGAACAGCTATG |
| --- | --- | --- |
| LDHA-HRE-1 | CTGACTGACTGCTAGGCATT | GACGACCTTCAGTTTCCTCATC |
| LDHA-HRE-2 | GGGAAGGAGAGCCACAAAG | CACCAGAGATACGTGCAGAAA |
| LDHA-intergenic | TCCTTCTTCCTCAGCCTCTTA | TGACAACTCACACCTGTTATCC |

**Table S4: Human Patients’ details**

| **Patient ID** | **SEX/Age** | **Histological Grade** | **Type of Cancer** | **TNM staging** |
| --- | --- | --- | --- | --- |
| MRU-18 | F/50 | Grade-2 | Invasive Ductal carcinoma with no special type. | pT_1c_N_1q_ |
| MRU-25 | F/60 | Grade-2 | Invasive Ductal carcinoma with no special type. | ypT_4b_N_1a_ |
| MRU-30 | F/54 | Grade-2 | Invasive Ductal carcinoma with no special type. | pT_2_N_1a_ |
| MRU-31 | F/49 | Grade-2 | Invasive Ductal carcinoma with no special type. | pT_4b_N_2a_ |
